# Supplementary material for: An improved approach to generate IL-15+/+/TGFβR2−/− iPSC-derived natural killer cells using TALEN
Source: Cell Rep Methods. 2024 Sep 10;4(9):100857. doi: 10.1016/j.crmeth.2024.100857 (PMC11440057; doi:10.1016/j.crmeth.2024.100857)
Supplement: Document S1. Figures S1–S4 [file mmc1.pdf]

Cell Reports Methods, Volume *4*

Supplemental information

An improved approach to generate IL-15$^{+/+}$/TGFβR2$^{-/-}$ iPSC-derived natural killer cells using TALEN

An-Ping Chen, Peng Gao, Liang Lin, Preeti Ashok, Hongzhi He, Chao Ma, David Li Zou, Vincent Allain, Alex Boyne, Alexandre Juillerat, Philippe Duchateau, Armin Rath, Daniel Teper, Antonio Arulanandam, Hao-Ming Chang, Justin Eyquem, and Wei Li

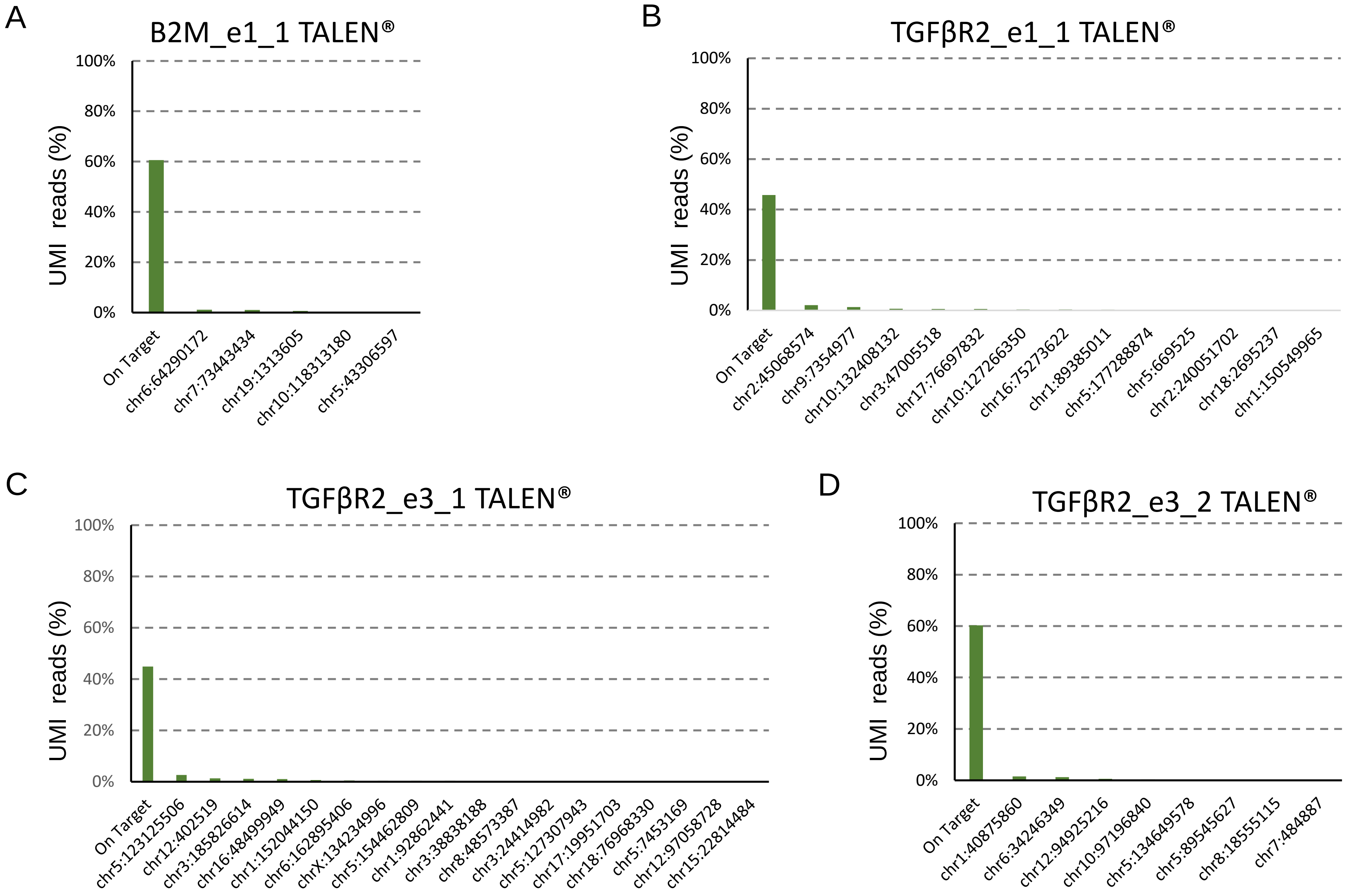

Figure S1: Potential candidate off-target site profiles of TALENs® targeting B2M and TGFbR2 loci in human iPSCs. Related to Table 1 and Table 2. Genome-wide potential candidate off-target sites for TALEN®s, B2M_e1_1, TGFβR2_e1_1, TGFβR2_e3_1 and TGFβR2_e3_2, in human iPSCs were analyzed by Guide-seq. The UMI (unique molecular identifier) reads percentage of the on-target site and potential candidate off-target sites for B2M_e1_1 TALEN® (A), TGFβR2_e1_1 TALEN® (B), TGFβR2_e3_1 TALEN® (C), and TGFβR2_e3_2 TALEN® (D) are displayed.

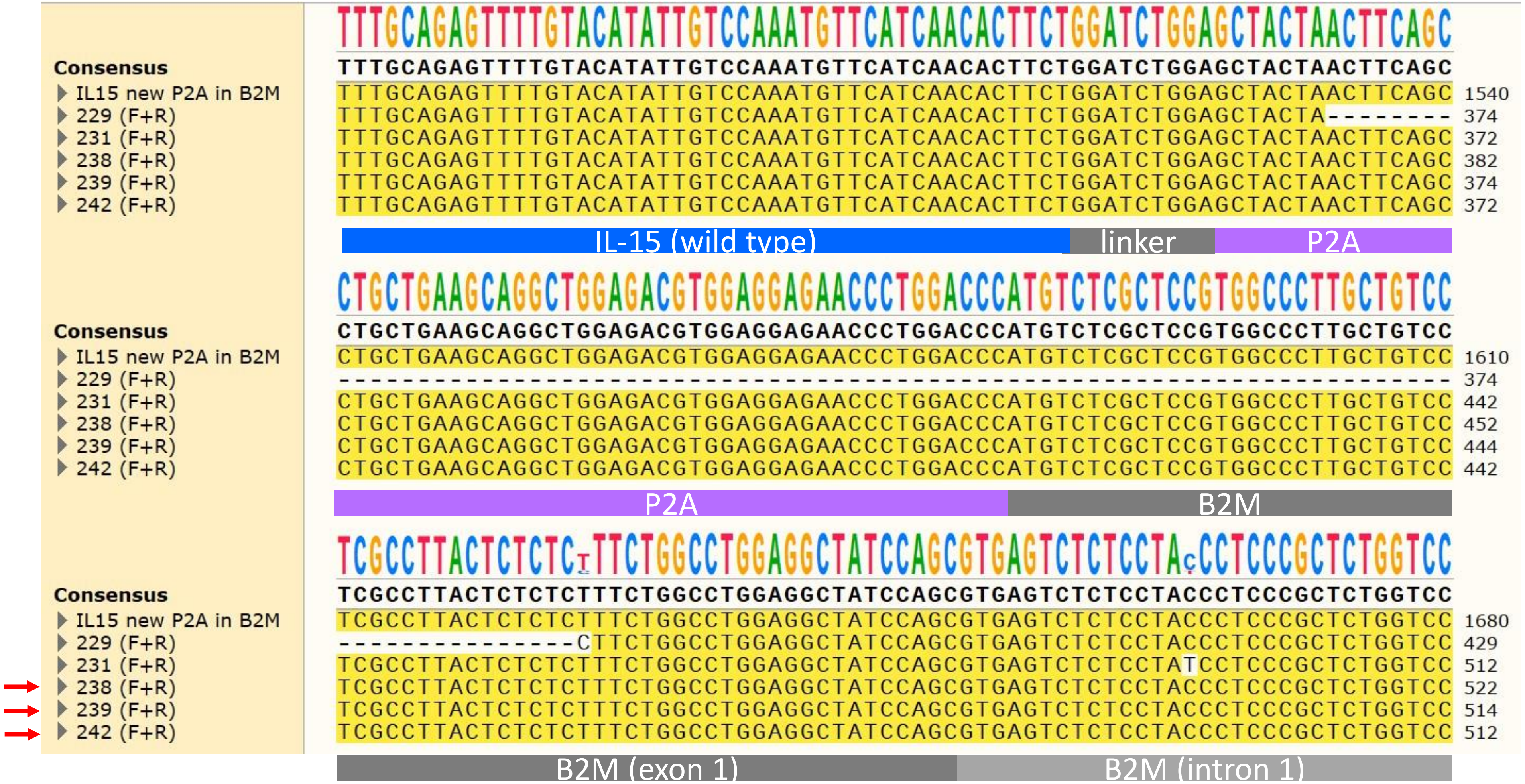

Figure S2: Precision of the insertion of IL15-P2A cassette at the *B2M* locus. Related to Figure 2. The PCR bands from five bi-allelic Knock-in iPSC clones (Figure 2F) were cut, purified, and sent for Sanger sequencing. Three of them (red arrow indicated) show desired knock-in sequence that encode the exogenous IL15 and the endogenous B2M exon1 in the same open reading frame (ORF).

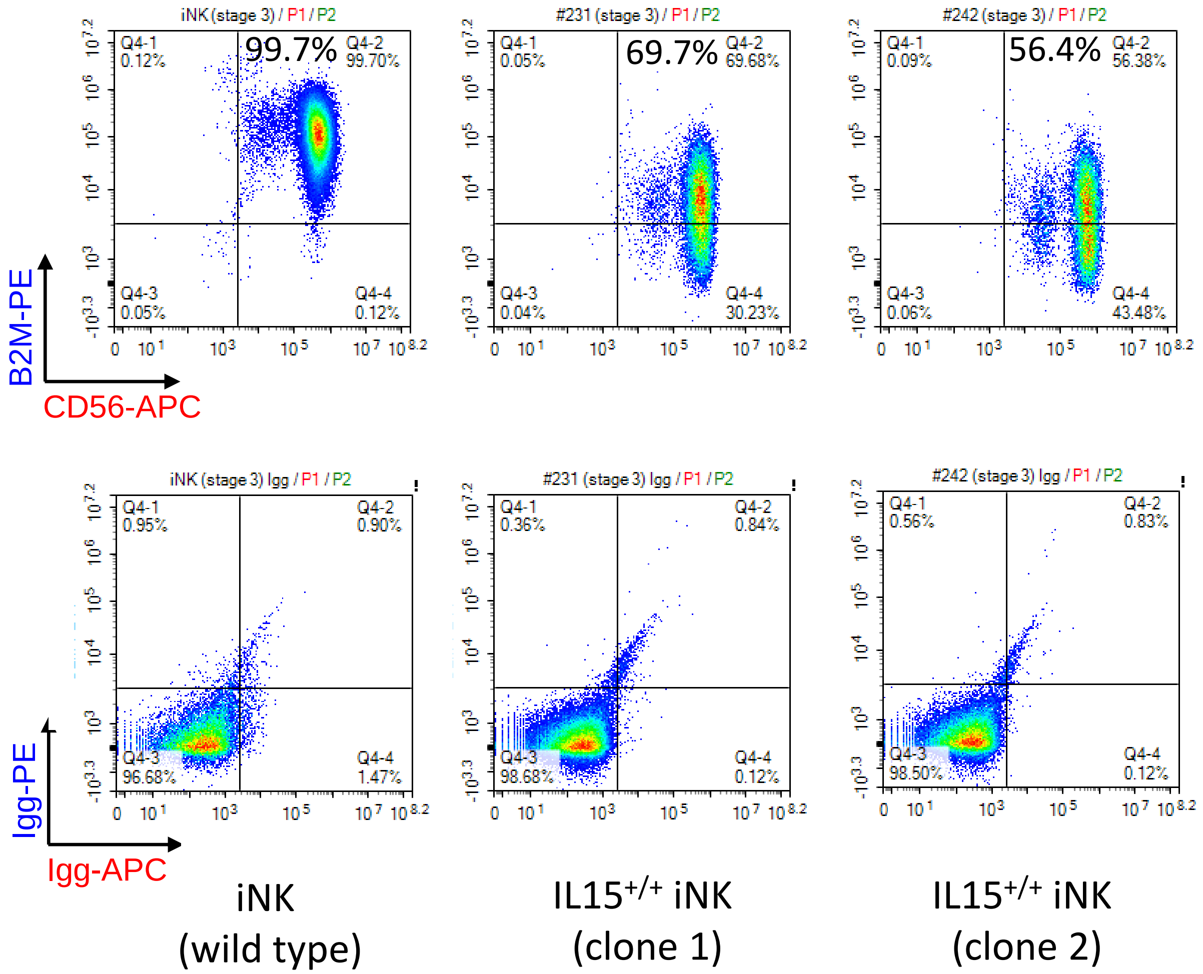

Figure S3: The expression of B2M were rescued in the IL15$^{+/+}$ iNK cells. Related to Figure 2. Flow-cytometry plots showing the surface expression level of B2M and CD56 in WT iNK cells and iNK cells from two IL15$^{+/+}$ iPSC clones (top panels). The flow-cytometry plots of control staining by Igg-PE and Igg-APC were also displayed (bottom panels).

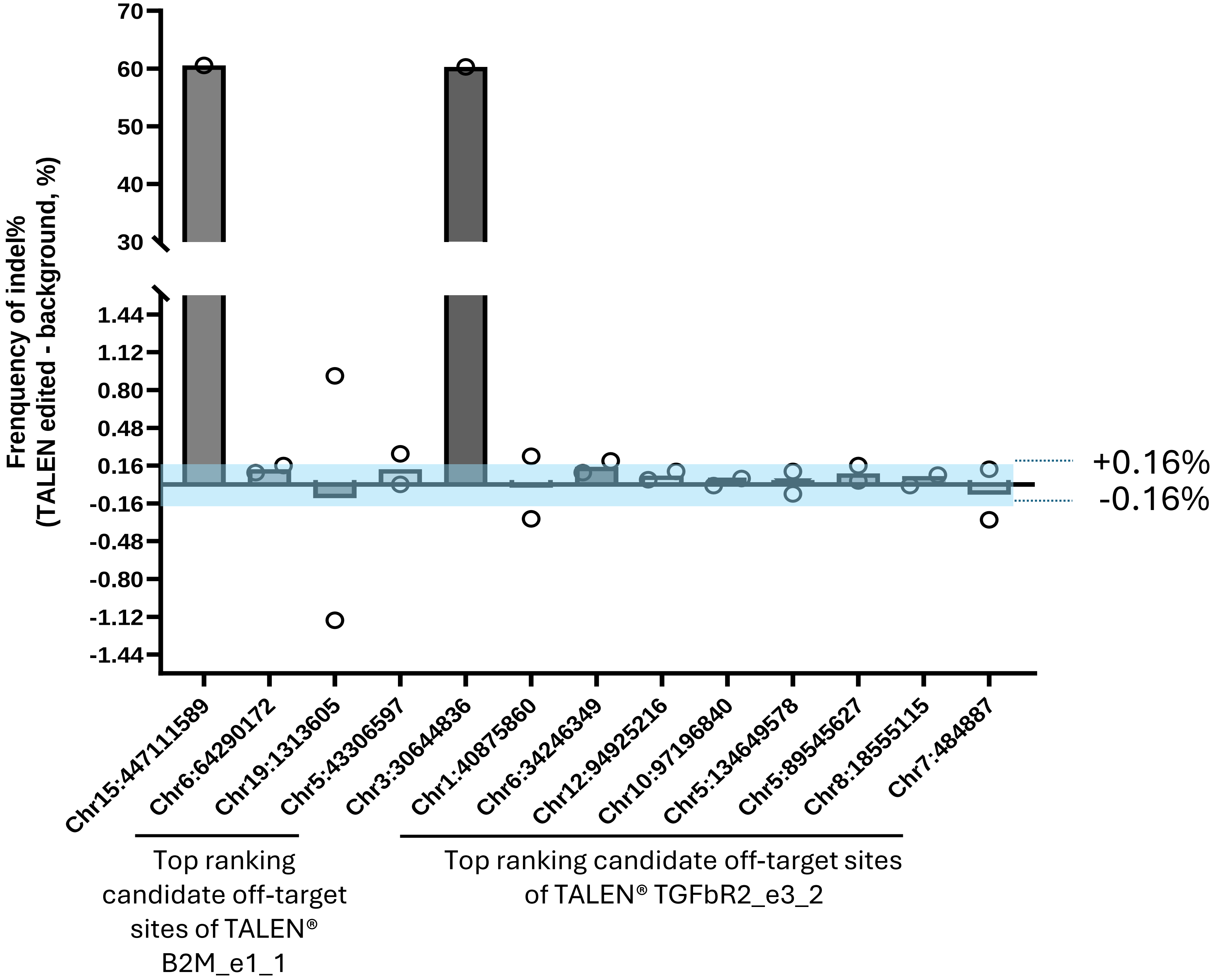

Figure S4: Validation/invalidation of potential candidate off-target sites in IL15$^{+/+}$/TGFβR2$^{-/-}$ iPSCs. Related to Table 1 and Table 2. Frequencies of insertion and deletions (indels) identified by Amplicon sequencing for the top candidate off-target sites of B2M_e1_1 and TGFβR2_e3_2 TALEN®s are displayed, except for two top-ranking candidate off-target sites of B2M_e1_1 TALEN® - chr7:73443435 and chr10:118313180 (see the explanation in the results). Frequencies of indels obtained in two individual IL15$^{+/+}$/TGFβR2$^{-/-}$ iPSC single clones subtracted from those obtained from the parent wild type iPSCs are indicated. The blue area indicates the data points falling within the threshold of significant indels detection (±0.16%). Frequencies of indels identified by GUIDE-seq for the on-sites of B2M_e1_1 and TGFβR2_e3_2 TALEN®s are also displayed in column 1 and 5.
