# Supplementary material for: An improved approach to generate IL-15+/+/TGFβR2−/− iPSC-derived natural killer cells using TALEN
Source: Cell Rep Methods. 2024 Sep 10;4(9):100857. doi: 10.1016/j.crmeth.2024.100857 (PMC11440057; doi:10.1016/j.crmeth.2024.100857)
Supplement: Document S2. Article plus supplemental information [file mmc2.pdf]

Cell Reports
Methods

Article

An improved approach to generate IL-15$^{+/+}$/TGFβR2$^{-/-}$ iPSC-derived natural killer cells using TALEN

Graphical abstract

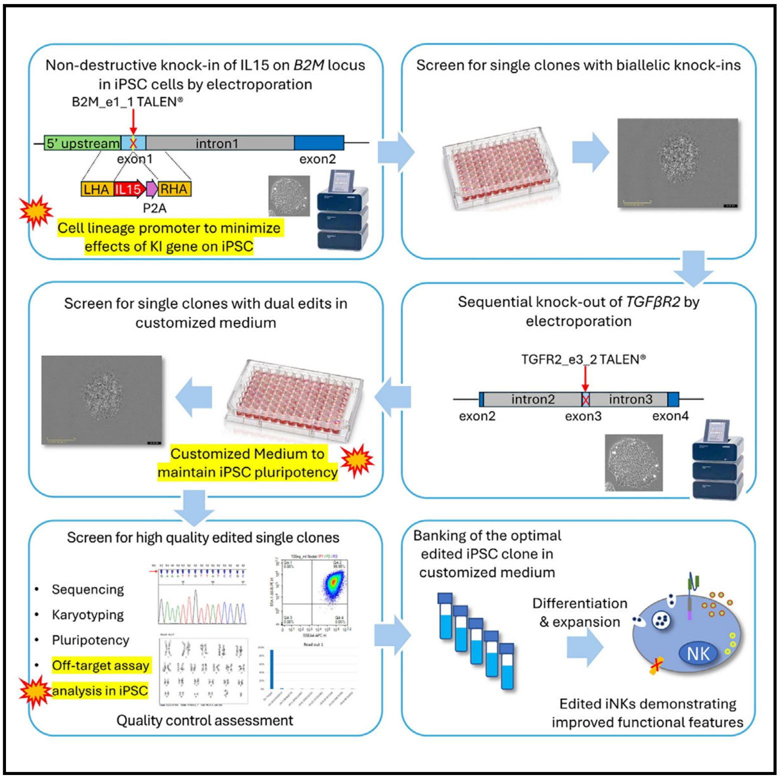

Highlights

- We present a TALEN-based workflow to generate dual gene-edited iPSC-derived NK cells
- It uses cell lineage promoter for expression of the knockin gene to minimize effects on iPSCs
- It uses customized medium to maintain pluripotency of iPSCs with disrupted TGF-β signaling
- Dual-edited iNK cells show improved persistence and enhanced functional characteristics

Authors

An-Ping Chen, Peng Gao, Liang Lin, ..., Hao-Ming Chang, Justin Eyquem, Wei Li

Correspondence

anping.chen@cytoviatx.com (A.-P.C.),
wei.li@cytoviatx.com (W.L.)

In brief

Chen et al. develop an improved method where soluble IL-15 is non-destructively integrated into iPSC under the endogenous B2M promoter, which has minimum activity in iPSC. They further optimize the culture medium for TGFβR2$^{-/-}$ iPSCs. The resulting IL-15$^{+/+}$/TGFβR2$^{-/-}$ iNK cells demonstrate improved persistence and function.

Check for updates

Chen et al., 2024, Cell Reports Methods *4*, 100857
September 16, 2024 © 2024 The Authors. Published by Elsevier Inc.
https://doi.org/10.1016/j.crmeth.2024.100857

CellPress

Cell Reports Methods

CellPress
OPEN ACCESS

Article

An improved approach to generate IL-15$^{+/+}$/TGFβR2$^{-/-}$ iPSC-derived natural killer cells using TALEN

An-Ping Chen,[1,8,*] Peng Gao,[1,8] Liang Lin,[1,8] Preeti Ashok,[1] Hongzhi He,[1] Chao Ma,[1] David Li Zou,[1] Vincent Allain,[5,6,7] Alex Boyne,[3] Alexandre Juillerat,[3] Philippe Duchateau,[4] Armin Rath,[2] Daniel Teper,[2] Antonio Arulanandam,[1] Hao-Ming Chang,[1] Justin Eyquem,[5,6] and Wei Li[1,9,*]
[1]Cytovia Therapeutics, Inc., Natick, MA, USA
[2]Cytovia Therapeutics, Inc., Aventura, FL, USA
[3]Cellectis Inc, New York, NY, USA
[4]Cellectis, Paris, France
[5]Gladstone-University of California, San Francisco Institute of Genomic Immunology, San Francisco, CA, USA
[6]Department of Medicine, University of California, San Francisco, San Francisco, CA, USA
[7]Université Paris Cité, INSERM UMR976, Hôpital Saint-Louis, Paris, France
[8]These authors contributed equally
[9]Lead contact
*Correspondence: anping.chen@cytoviatx.com (A.-P.C.), wei.li@cytoviatx.com (W.L.)
https://doi.org/10.1016/j.crmeth.2024.100857

MOTIVATION Gene-edited iPSC-derived immune cells are an attractive vehicle for cancer immunotherapy; however, the expression of exogenous genes may impact their pluripotency and potential for differentiation. To establish a robust gene-edited iPSC-derived immune cell therapy platform, we used a promoter directing minimal expression of the knockin (KI) gene in iPSCs while promoting increased expression of the exogenous gene in differentiated iPSC-derived immune cells. In addition, we developed a customized iPSC culture medium that preserves the pluripotency of iPSC with TGFβR2 KO, which facilitates the production of master cell banks of the edited iPSC clones.

SUMMARY

We present a TALEN-based workflow to generate and maintain dual-edited (IL-15$^{+/+}$/TGFβR2$^{-/-}$) iPSCs that produce enhanced iPSC-derived natural killer (iNK) cells for cancer immunotherapy. It involves using a cell lineage promoter for knocking in (KI) gene(s) to minimize the potential effects of expression of any exogenous genes on iPSCs. As a proof-of-principle, we KI IL-15 under the endogenous B2M promoter and show that it results in high expression of the sIL-15 in iNK cells but minimal expression in iPSCs. Furthermore, given that it is known that knockout (KO) of TGFβR2 in immune cells can enhance resistance to the suppressive TGF-β signaling in the tumor microenvironment, we develop a customized medium containing Nodal that can maintain the pluripotency of iPSCs with TGFβR2 KO, enabling banking of these iPSC clones. Ultimately, we show that the dual-edited IL-15$^{+/+}$/TGFβR2$^{-/-}$ iPSCs can be efficiently differentiated into NK cells that show enhanced autonomous growth and are resistant to the suppressive TGF-β signaling.

INTRODUCTION

Natural killer (NK) cells have the innate ability to recognize and spontaneously eliminate "stressed" cells, such as tumor cells, without prior priming. Recent clinical trials have shown that NK cells can effectively target acute myeloid leukemia,[1,2] gastrointestinal stromal tumors,[3] ovarian cancer,[4] and breast cancer[5] without inducing adverse side effects like graft-versus-host disease, neurotoxicity, or cytokine release syndrome.[6,7] However, NK cells are usually short-lived, and the clinical efficacy of NK cells is often compromised by their limited persistence as well as tumor-intrinsic immunosuppressive microenvironments where transforming growth factor β (TGF-β) plays a central role in inhibiting NK cell functions. Meanwhile, overexpression of soluble interleukin-15 (sIL-15),[8] membrane-bound IL-15 (mIL-15),[9] or IL15-IL15Rα complex,[10] together with chimeric antigen receptors (CARs) has demonstrated enhanced anti-tumor efficacy and persistence *in vitro* and *in vivo*. Therefore, we hypothesized that

© 2024 The Authors. Published by Elsevier Inc.
This is an open access article under the CC BY license (http://creativecommons.org/licenses/by/4.0/).

induced pluripotent stem cell (iPSC)-derived NK cells, with an IL-15 knockin (KI) and TGFβR2 knockout (KO) could exhibit improved immune function and overcome the immunosuppressive tumor microenvironment (TME).

For allogeneic immune cell therapies, there are two major sources for the cells—one is a donor-derived approach and the other is an iPSC-derived approach. With their unlimited self-renewal capability, iPSCs, as a starting material, can minimize the donor-to-donor and batch-to-batch variations that face the donor-derived approach. In addition, the biggest advantage of the iPSC-derived approach is in gene editing. This is because, with the iPSC-derived approach, the gene editing is performed at the iPSC level with a small number of cells, followed by screening for gene-edited iPSC single-cell clones with rigorous quality control (QC) characterizations. After an optimally gene-edited single iPSC clone is selected, a master cell bank (MCB) of the clone will be produced to serve as the source of starting material for subsequent differentiation and expansion. Therefore, unlike a donor-derived approach where gene editing needs to be performed for each batch, with an iPSC-derived approach, gene editing is a one-time event for each edited product. More important, with an iPSC-derived approach, the final edited immune cell product is homogeneously gene edited and therefore is consistent throughout the development cycle since all the edited immune cells are derived from a single iPSC clone. In contrast, with the donor-derived approach, the final product consists of a heterogeneously gene-edited cell population, and hence, there can be large batch-to-batch variations. This advantage of the iPSC-derived approach amplifies when multiple rounds of gene editing are needed.

Lentiviral delivery methods utilizing constitutive promoters, such as elongation factor 1α (EF1α) or cytomegalovirus (CMV) promoter, have been widely used for the insertion of exogenous genes randomly into human iPSCs.[11] Recently, a highly efficient and precise transgene KI technique, also under a strong constitutive promoter, was applied to human iPSCs, targeting a site within an exon of an essential gene, while preserving the essential gene's function.[12] However, concerns have arisen regarding the potential unforeseen effects of high exogenous gene expression on the expansion and differentiation of human iPSCs.[13] Therefore, we set out to explore expressing exogenous genes under cell lineage promoters that have no/minimal activity in iPSC for generation of our edited iPSC-derived NK (iNK) cells. Even though the B2M gene has long been considered a housekeeping gene, it is actually minimally expressed in iPSCs,[14] despite high expression in NK cells and many other cell types. Thus, we consider its promoter to be a cell lineage promoter, rather than a housekeeping promoter. Hence, we developed an improved method to take advantage of the cell lineage characteristic of the B2M promoter, whereby the soluble IL-15 is precisely integrated under the endogenous promoter of B2M without disrupting the expression of B2M.

Furthermore, TGF-β plays a key role in maintaining the pluripotency of iPSCs, and therefore the pluripotency of iPSCs with TGFβR2 KO in normal iPSC culture medium cannot be sustained beyond 2 weeks due to the disruption of the TGF-β signaling pathway.[15] We therefore developed a customized iPSC medium containing Nodal and demonstrated that this customized medium can adequately preserve the pluripotency of iPSCs with TGFβR2 KO.

With the above two improvements, we generated IL15$^{+/+}$/TGFβR2$^{-/-}$ iPSCs using TALEN. The resulting morphology- and karyotyping-normal, dual-edited iPSC single clones were then differentiated into NK cells with high efficiency. These edited iNK cells persisted longer than unedited iNK cells in the absence of exogenous cytokines and demonstrated resistance to the suppressive TGF-β signaling.

RESULTS

Utilizing the endogenous B2M promoter for KI in iPSCs

Gene-edited iPSCs hold significant promise for drug discovery, disease modeling, and personalized medicine. However, the high expression of exogenous genes under a constitutive promoter may affect the quality of iPSCs, such as its pluripotency and differentiation efficiency. In contrast, cell lineage promoters have low/no activity in stem cells while having increased activity during or after the course of stem cell differentiation. The B2M promoter is such a cell lineage promoter since it has been reported that the expression of B2M is minimal in iPSCs,[14] whereas it is very high in NK cells.

To examine and confirm the expression pattern of the B2M gene, we conducted qRT-PCR using glyceraldehyde 3-phosphate dehydrogenase (GAPDH) as an internal reference and compared it to a well-known NK-specific gene, NKG2A. The relative expression levels of B2M and NKG2A are depicted in Figures 1A and 1B. In iPSCs, B2M exhibits a minimal expression level (1.8% of GAPDH's expression), while NKG2A expression is undetectable (Figures 1A and 1B). However, B2M shows a nearly 3-fold higher expression level than GAPDH in iNK cells and peripheral blood NK (PBNK) cells, while their expression levels are very similar in cord blood NK (CBNK) cells. In contrast, NKG2A demonstrates lower expression levels (only 7.5%–11.5% of the GAPDH expression levels) in iNK, CBNK, and PBNK cells. Based on these results, we selected the endogenous B2M promoter to drive the expression of exogenous gene(s) in edited iPSC-derived NK cells to minimize the impact of the exogenous gene(s) on iPSC. Although NKG2A expression is undetectable in iPSCs, its expression levels in NK cells are also low, and therefore the endogenous NKG2A promoter may not be a good choice for our purpose.

Design and screening of TALENs for gene editing in iPSCs

To position IL-15 under B2M's robust endogenous promoter, we designed and generated TALEN B2M_e1_1 to target the first exon of B2M (Figure 1D). The left and right TALEN binding sequences consists of 17 bp each, with a 13-bp spacer (Figure 2A). Amplicon sequencing was performed to assess the on-target gene-editing efficiency of B2M e1_1 TALEN in iPSCs, and we observed that within the electroporated cell population, 94.99% of the alleles exhibited mutations (deletion or insertion), with a frame-shifting percentage of 76.89% (Figure 2E).

In addition, B2M, a component of major histocompatibility complex class I, functions as a self-recognition marker on the surface of NK cells. Previous reports indicate that disruption of

CellPress
OPEN ACCESS

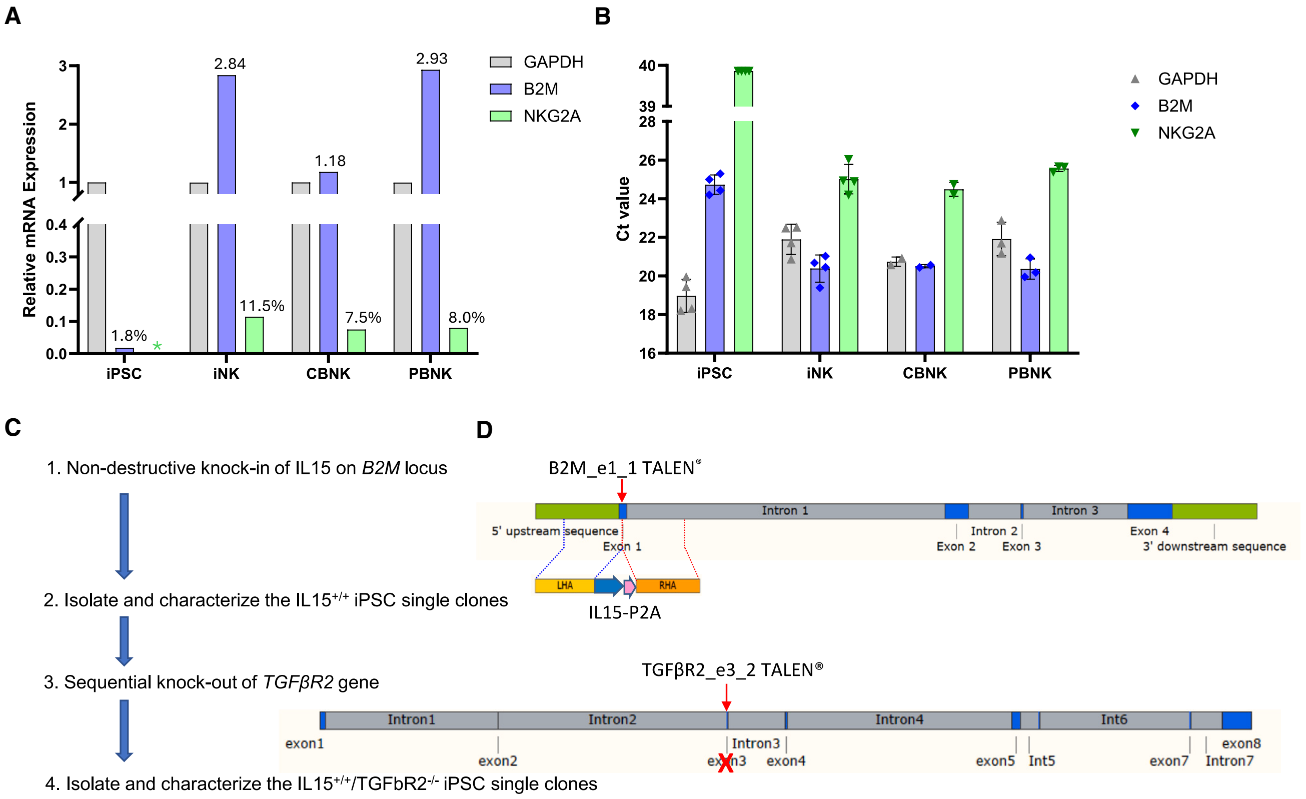

Figure 1. Expression pattern of B2M and the strategy of design and generation of IL-15$^{+/+}$/TGFβR2$^{-/-}$ iPSC single clones

(A) Relative expression levels of B2M and NKG2A, with GAPDH as an internal reference in iPSC, iNK, CBNK, and PBNK cells. Asterisk indicates undetectable. The relative expression level was calculated by the formula $2^{\Delta Ct}$.

(B) Mean Ct values (mean ± SE) of GAPDH, B2M, and NKG2A genes in qPCR in iPSC, iNK, CBNK, and PBNK cells. Two to four samples of different cell types were used in this experiment.

(C) The workflow of generation of IL15$^{+/+}$/$^{-/-}$ iPSC clones by sequential gene editing with TALEN.

(D) Schematic diagram of non-destructive KI of IL-15 at *B2M* exon1 by B2M_e1_1 TALEN, design of the linearized IL-15 donor plasmid, and disruption of TGFβR2 at *TGFβR2* exon 3 by TGFβR2e3_2 TALEN.

the B2M gene leads to the fratricide of NK cells.[16] Therefore, to take advantage of the endogenous B2M promoter while preventing NK cell fratricide due to loss of B2M, we designed a donor plasmid encoding a soluble form of IL-15, followed by a self-cleaving P2A peptide as a linker as a way to put the sIL-15 and the endogenous B2M gene within the same open reading frame (Figure 1D).

For knocking out the *TGFβR2* gene, we designed and generated three TALENs: TGFβR2_e1_1, TGFβR2_e3_1, and TGFβR2_e3_2. TGFβR2_e1_1 targets exon 1, while TGFβR2_e3_1 and TGFβR2_e3_2 target exon3 sequences of the *TGFβR2* gene. The left and right TALEN binding sequences consist of 17 bp, while the spacer is either 13 or 14 bp (Figures 2B–2D). Amplicon sequencings were also performed to assess the on-target gene-editing efficiencies of these three *TGFβR2* TALENs in iPSCs. The percentages of mutated alleles within the electroporated cell population are 93.96% for TGFβR2_e1_1, 23.72% for TGFβR2_e3_1, and 67.68% for TGFβR2_e3_2 (Figure 2E; Table 1). Additionally, the frame-shifting percentages are 48.4% for TGFβR2_e1_1, 17.86% for TGFβR2_e3_1, and 56.12% for TGFβR2_e3_2 TALENs.

Next, we investigated the candidate off-target sites for B2M_e1_1, TGFβR2_e1_1, TGFβR2_e3_1, and TGFβR2e3_2 TALENs using genome-wide unbiased identification of double-stranded breaks enabled by sequencing (GUIDE-seq).[17] GUIDE-seq, known for its high validation rate compared to most *in vitro* methods, has become a gold standard for genome-wide analysis of off-target sites in primary cells[18] and other cell lines. However, the candidate off-target sites identified by GUIDE-seq need to be assessed in the actual gene-edited cells by amplicon sequencing to validate whether the off-target gene-edits indeed occurred at these sites. Five candidate off-target sites were identified for the B2M_e1_1 TALEN (Figure S1A). The highest percentage of unique molecular identifier (UMI) reads of those candidate off-target sites is 1.1246%. Notably, all candidate off-target sites are located in intronic or intergenic regions. The accurate reads and the related gene of each candidate off-target site of B2M_e1_1 TALEN are listed in Table 2. Thirteen candidate off-target sites were identified for TGFβR2_e1_1 TALEN (Figure S1B) and 18 candidate off-target sites were identified for TGFβR2_e3_1 TALEN (Figure S1C). Notably, among the three TGFβR2 TALENs, the lowest number of candidate off-target sites (only eight) were

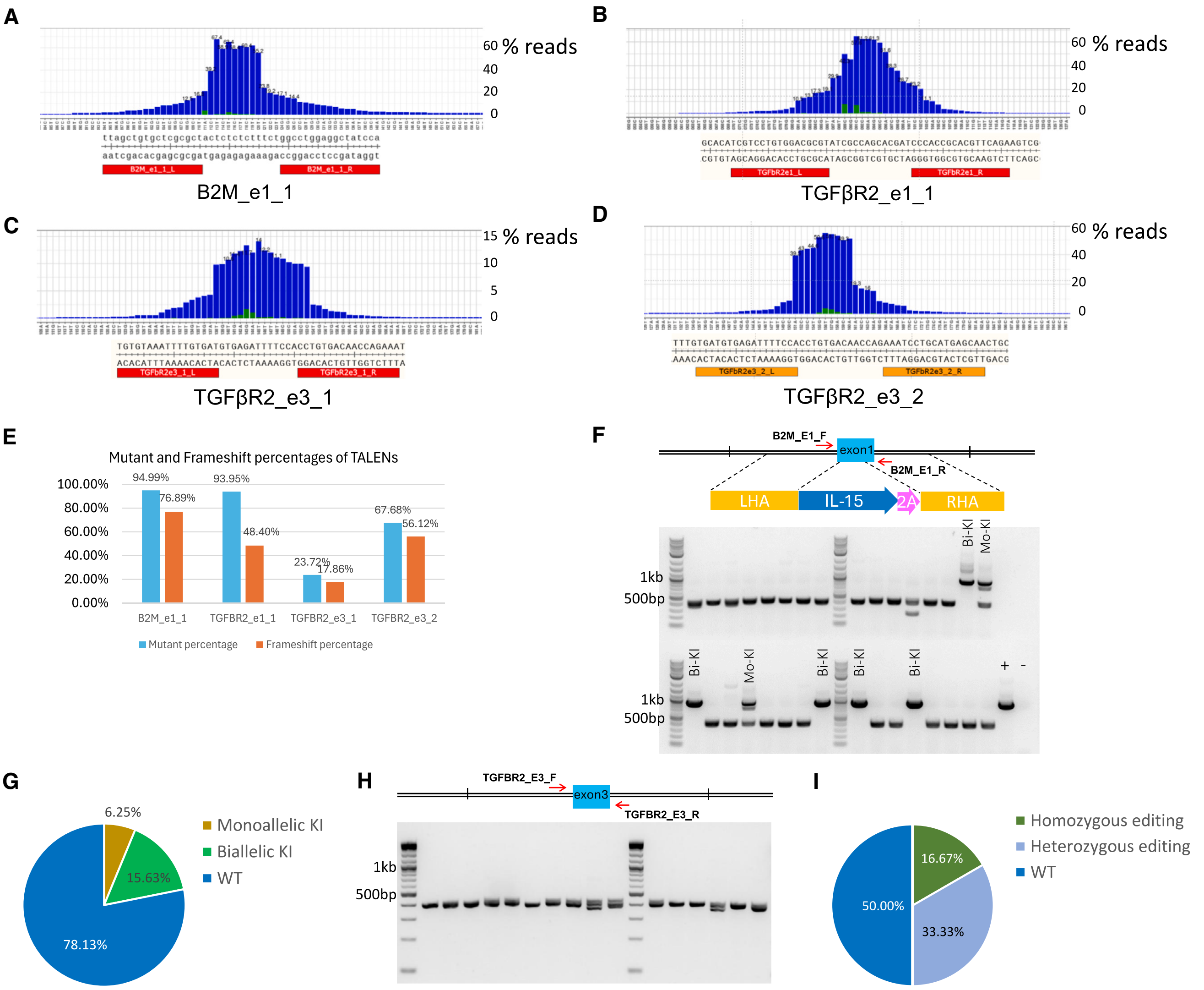

Figure 2. Genome-editing efficiencies for B2M_e1_1, TGFβR2_e1_1, TGFβR2_e3_1, and TGFβR2_e3_2 TALENs in human iPSC cells, and genotypic characterization of IL-15$^{+/+}$/TGFβR2$^{-/-}$ iPSC single clones

(A) Genomic DNA was extracted from the iPSCs electroporated with B2M_e1_1 TALEN. Then, the B2M exon1 locus was amplified by PCR. Amplicons were sent for Amplicon-EZ sequencing and analyzed to detect the indels. Indel distribution within individual nucleotides was displayed. The left and right TALEN-binding sites are indicated.

(B) Genomic DNA was extracted from the iPSC cells electroporated with TGFβR2_e1_1 TALEN. Then, the TGFβR2 exon1 locus was amplified by PCR. Indel distribution within individual nucleotides was displayed. The left and right TALEN binding sites are indicated.

(C) Genomic DNA was extracted from the iPSC cells electroporated with TGFβR2_e3_1 TALEN. Then, the TGFβR2 exon3 locus was amplified by PCR. Indel distribution within individual nucleotides was displayed. The left and right TALEN binding sites are indicated.

(D) Genomic DNA was extracted from the iPSC cells electroporated with TGFβR2_e3_2 TALEN. Then, the TGFβR2 exon3 locus was amplified by PCR. Indel distribution within individual nucleotides was displayed. The left and right TALEN binding sites are indicated.

(E) Mutant percentage and frameshift mutant percentage for B2M_e1_1, TGFβR2_e1, TGFβR2_e3_1, and TGFβR2_e3_2 TALEN in human iPSCs.

(F) Genomic DNAs of 32 candidate IL-15 KI iPSC single clones were extracted and were screened for KI by PCR. Top: the locations of the PCR primers for amplifying the KI cassette, IL-15-P2A, at the B2M locus; bottom: PCR products of B2M site-specific IL-15 KIs (Bi-KI, biallelic KI; Mo-KI, monoallelic KI) were displayed together with the positive control (+) and negative control (–) in the gel.

(G) A pie chart illustrating the proportions of monoallelic KI, biallelic KI, and the WT among 32 candidate iPSC clones.

(H) The genomic DNA of 16 candidate IL-15$^{+/+}$/TGFβR2$^{-/-}$ iPSC single clones was extracted and screened for TGFβR2 KO by PCR. Top: the locations of the PCR primers for amplifying the editing region at the TGFβR2 locus; bottom: the amplified bands were displayed in the gel. The PCR bands were purified and further characterized by Sanger sequencing.

(I) A pie chart illustrating the proportions of homozygous KO, heterozygous KO, and the WT among 12 selected iPSC clones.

Table 1. Mutant percentage and frameshifting percentage characterized by amplicon sequencing for B2M_e1_1 TALEN and three TGFβR2 TALENs in human iPSC cells

Sample	B2M e1_1	TGFβR2_e1_1	TGFβR2_e3_1	TGFβR2_e3_2
Target reads	240,232	259,493	455,104	228,629
Mutant reads	228,188	243,822	107,933	154,747
Mutant percentage	94.99	93.96	23.72	67.68
Frameshift mutant reads	184,706	125,598	81,290	128,313
Frameshift mutant percentage	76.89	48.4	17.86	56.12

identified for TGFβR2_e3_2 TALEN (Figure S1D). Detailed information, including accurate reads and the related genes of each candidate off-target site for the three TGFβR2 TALENs, is shown in Table 2. The maximum percentage of UMI reads for the candidate off-target sites for TGFβR2_e1_1 TALEN is 2.0682%. Except for one candidate off-target site, chr3:47005518, which is in the exon of the *NBEAL2* gene and has a potential of 0.5536%, the remaining sites are situated in the 5′ UTR, intron, or intergenic regions (Table 2, TGFβR2_e1_1 TALEN category). In the case of TGFβR2_e3_1 TALEN, the highest percentage of UMI reads among the candidate off-target sites is 2.6042%. Except for one candidate off-target site, chr18:76968330, which is located in the exon of the *ZNF236* gene and has a potential of 0.1403%, the rest are located in the intron or intergenic regions (Table 2, TGFβR2_e3_1 TALEN category). For the candidate off-target sites for TGFβR2_e3_2 TALEN, the maximum UMI read percentage is 1.5423%. All eight of these candidate off-target sites are in downstream, intron, or intergenic regions (Table 2, TGFβR2_e3_2 TALEN category). Given that TGFβR2_e3_2 TALEN exhibits the highest frameshift percentage at the *TGFβR2* locus and the lowest potential candidate off-target sites in human iPSCs, we selected it along with the B2M_e1_1 TALEN for generating dual gene-edited iPSCs in the subsequent experiments.

Generation of IL-15$^{+/+}$/TGFβR2$^{-/-}$ dual-edited iPSC clones

To generate the IL-15 KI iPSCs, IL-15 donor plasmid and the B2M_e1_1 TALEN were co-electroporated into iPSCs, followed by sorting the electroporated iPSCs for single clones in a 96-well plate using a single-cell dispenser. A total of 32 single clones were obtained and screened through PCR and then Amplicon sequencing. Among these clones, monoallelic KIs (IL-15$^{+/-}$) and biallelic KIs (IL-15$^{+/+}$) accounted for 6.25% and 15.63% of the screened clones, respectively (Figures 2F and 2G).

Importantly, the non-destructive KI strategy preserves the expression of the B2M gene. We verified the junction region between the IL-15-P2A and the B2M exon1 in edited IL-15$^{+/+}$ iPSCs through Sanger sequencing (Figure S2). Subsequently, we investigated the B2M expression in edited IL-15$^{+/+}$ iNK cells, derived from two edited IL-15$^{+/+}$ iPSC clones. Our results confirmed the expression of B2M in these edited cells, even though the expression levels are lower than non-edited iNK cells (Figure S3).

From our pool of single clones harboring a biallelic IL-15 KI (IL-15$^{+/+}$) with confirmed precise insertion, most of them have normal karyotypes. We cultured one of these clones and subsequently electroporated the cells with TGFβR2_e3_2 TALEN to knock out *TGFβR2* alleles. The electroporated IL-15$^{+/+}$ iPSC cells were then cultured and sorted in a 96-well plate using a single-cell dispenser to generate individual clones (Figure 2H). KO events were identified through PCR and then amplicon sequencing. Among the 16 candidate single clones, 12 displayed 1 PCR band, suggesting that these clones may be homozygous KOs. These PCR bands were purified and sent for Sanger sequencing. The sequencing chromatograms from four single clones revealed double peaks, indicative of heterozygous editing at their *TGFβR2* locus. However, the sequencing results for two single clones (16.67% of total) show homozygous editing, one with a 10-bp deletion and the other with a 14-bp deletion, both resulting in frameshifts (Figure 2I). These two single clones represent biallelic IL-15 KI and homozygous TGFβR2 KO iPSC clones (IL-15$^{+/+}$/TGFβR2$^{-/-}$).

Specificity analyses of TALEN B2M_e1_1 and TGFbR2_e3_2 in IL-15$^{+/+}$/TGFβR2$^{-/-}$ iPSC clones

Even though we have conducted GUIDE-seq assays for the two TALENs used to generate IL-15$^{+/+}$/TGFβR2$^{-/-}$ iPSC clones, which allowed us to unbiasedly identify candidate off-target sites in human iPSCs, to verify whether these potential off-target edits actually happen in the edited iPSC clones, amplicon sequencing assays were performed for the two IL-15$^{+/+}$/TGFβR2$^{-/-}$ iPSC clones and compared the results with the parent non-edited iPSC clone.

We found that the mutant percentage of two candidate off-target sites of TALEN B2M_e1_1 (chr7:73443435 and chr10:118313180) are ~50% in both IL-15$^{+/+}$/TGFβR2$^{-/-}$ iPSC clones and the parent non-edited iPSC clone. After further investigation, the chr7:73443435 site was found neighboring the existing SNV—rs1583880367 (A = 0.5, C = 0.5). This SNV is located in the intron of the BAZ1B gene. The other candidate off-target site, chr10:118313180, neighbors to a 13 consecutive adenine (A). A high percentage of insertions/deletions (indels) were detected in this region. During the Sanger sequencing, poor data were often seen following a stretch of mononucleotides due to the disassociation and rehybridization in a different location. Based on the above reasons, we removed those two sites from further analysis.

The indel frequencies of the other 11 top-ranking candidate off-target sites for the two TALENs fall below the threshold of relevant detection[19] (threshold ±0.16%, described in the STAR Methods) (Figure S4), suggesting that sequential editing by B2M_e1_1 TALEN and the TGFβR2_e3_2 TALEN does not induce any significant off-target editing.

Table 2. The on-target and potential candidate off-target sites for B2M_e1_1, TGFβR2_e_1_1, TGFβR2_e_3_1, and TGFβR2_e_3_2 TALENs identified by GUIDE-seq in human iPSC cells

Chr	Position	UMI reads	Percentage	Gene	Annotation
B2M_e1_1 TALEN					
Chr15	447111589	355,157	60.5600	B2M	exon
Chr6	64290172	6,595	1.1246	EYS	intron
Chr7	73443435	6,000	1.0231	BAZ1B	intron
Chr19	1313605	3,819	0.6512	N.A.	intergenic region
Chr10	118313180	1,407	0.2399	FAM204A	intron
Chr5	43306597	993	0.1693	HMGCS1	intron
TGFβR2_e_1_1 TALEN					
Chr3	30606949	365,022	45.7763	TGFβR2	exon
Chr2	45068574	16,489	2.0682	N.A.	intergenic region
Chr9	7354977	10,390	1.3032	N.A.	intergenic region
Chr10	132408132	5,391	0.6761	PWWP2B	intron
Chr3	47005518	4,414	0.5536	NBEAL2	exon
Chr17	76697832	4,154	0.5210	MXRA7	intron
Chr10	127266350	3,057	0.3834	DOCK1	intron
Chr16	75273622	2,812	0.3527	N.A.	intergenic region
Chr1	89385011	2,359	0.2958	GBP6	intron
Chr5	177288874	1,111	0.1393	NSD1	exon
Chr5	669525	981	0.1230	TPPP	intron
Chr2	240051702	852	0.1068	OR6B3	intron
Chr18	2695237	635	0.0796	SMCHD1	intron
Chr1	150549965	527	0.0661	ADAMTSL4	5′ UTR
TGFβR2_e_3_1 TALEN					
Chr3	30644825	324,986	44.9112	TGFβR2	exon
Chr5	123125506	18,840	2.6042	PRDM6	intron
Chr12	402519	9,619	1.3296	CCDC77	intron
Chr3	185826614	7,892	1.0908	N.A.	intergenic region
Chr16	48499949	7,370	1.0187	N.A.	intergenic region
Chr1	152044150	4,662	0.6444	N.A.	intergenic region
Chr6	162895406	3,324	0.4594	PACRG	intron
ChrX	134234996	3,067	0.4239	N.A.	intergenic region
Chr5	154462809	2,490	0.3441	N.A.	intergenic region
Chr1	92862441	2,461	0.3401	DIPK1A	intron
Chr3	38838188	1,962	0.2712	N.A.	intergenic region
Chr8	48573387	1,611	0.2226	N.A.	intergenic region
Chr3	24414982	1,577	0.2179	THRB	intron
Chr5	127307943	1,336	0.1846	MEGF10	intron
Chr17	19951703	1,127	0.1557	AKAP10	intron
Chr18	76968330	1,015	0.1403	ZNF236	exon
Chr5	7453169	973	0.1344	ADCY2	intron
Chr12	97058728	792	0.1094	N.A.	intergenic region
Chr15	22814484	404	0.0558	NIPA1	intron
TGFβR2_e_3_2 TALEN					
Chr3	30644836	726,562	60.2861	TGFβR2	exon
Chr1	40875860	18,581	1.5423	N.A.	intergenic region
Chr6	34246349	15,266	1.2671	HMGA1	downstream
Chr12	94925216	7,597	0.6306	N.A.	intergenic region

(*Continued on next page*)

Table 2. *Continued*

Chr	Position	UMI reads	Percentage	Gene	Annotation
Chr10	97196840	4,052	0.3363	N.A.	intergenic region
Chr5	134649578	3,111	0.2582	SEC24A	intron
Chr5	89545627	1,630	0.1352	N.A.	intergenic region
Chr8	18555115	1,207	0.1001	PSD3	intron
Chr7	484887	572	0.0474	N.A.	intergenic region

N.A., not applicable.

Customizing culture medium for preserving pluripotency of TGFβR2$^{-/-}$ iPSC clones

It is well known that the TGF-β family signaling pathway plays a crucial role in regulating the delicate balance between self-renewal and differentiation in PSCs.[20] To gain deeper insight into the impact of TGFβR2 KO in human iPSCs, we cultured several TGFβR2 homozygous KO iPSC clones (TGFβR2$^{-/-}$) in regular E8 medium. As expected, we observed a dramatic decrease in the expression of classic pluripotency markers TRA-1-60 and SSEA4 after 2–3 weeks of culture (Figure 3A).

To overcome this issue, we cultured and passaged TGFβR2$^{-/-}$ iPSCs in E8 medium supplemented with varying concentrations of activin A or Nodal. After 18 days in the medium with activin A, the expression level of TRA-1-60 of TGFβR2$^{-/-}$ iPSCs slightly decreased to a range of 85.9%–93.2% (Figures 3B–3E). Simultaneously, the mean fluorescence intensity (MFI) of SSEA4 also decreased. Meanwhile, in the medium with Nodal, the double-positive population (TRA-1-60$^+$/SSEA4$^+$) of TGFβR2$^{-/-}$ iPSCs remained over 99.9% (Figures 3G–3J). To further confirm that Nodal supplement can preserve the pluripotency of iPSCs with TGFβR2 KO, we maintained these cells in the customized culture for more than 10 passages and observed that the pluripotency of these cells was sustained at the end of the experiment. These results demonstrate that this customized medium will pave the way for creating the MCB of iPSCs with TGFβR2 homozygous KO.

Characterization of IL-15$^{+/+}$/TGFβR2$^{-/-}$ dual-edited iPSC clones

The E8 medium supplemented with 100 ng/mL Nodal was used to culture and passage our IL-15$^{+/+}$/TGFβR2$^{-/-}$ dual-edited iPSCs. The flow cytometry analysis revealed that 99% of the IL-15$^{+/+}$/TGFβR2$^{-/-}$ iPSC cell population are double positive for these two markers (Figure 4A). This result confirmed that IL-15$^{+/+}$/TGFβR2$^{-/-}$ iPSC cells kept their pluripotency in our customized medium.

Microscopic examination of the IL-15$^{+/+}$/TGFβR2$^{-/-}$ iPSCs further supported their undifferentiated state. These cells displayed as compact colonies with distinct borders and well-defined edges (Figure 4B), which are characteristic features of undifferentiated iPSCs. Additionally, cytogenetic analysis was performed on 20 G-banded metaphase cells from the IL-15$^{+/+}$/TGFβR2$^{-/-}$ iPSC population. The results demonstrated an apparently normal male karyotype (46, XY) (Figure 4C).

The concentration of soluble IL-15 detected in the medium cultured with wild-type (WT) iPSCs or IL-15$^{+/+}$ iPSCs are 1–2 pg/mL per 1E6 live cells (data not shown) within 72 h post-medium change. There is no significant difference between the concentration of soluble IL-15 in the medium of WT iPSCs and that of IL-15$^{+/+}$ iPSCs, confirming the minimal activity of the B2M promoter in iPSCs.

Phenotyping of the IL-15$^{+/+}$/TGFβR2$^{-/-}$ iNK cells

The normal-karyotype IL-15$^{+/+}$/TGFβR2$^{-/-}$ iPSC single clones were then differentiated into IL-15$^{+/+}$/TGFβR2$^{-/-}$ iNK cells. We then expanded these edited iNK cells to a sufficient quantity for subsequent phenotypic analysis using flow cytometry. The histograms in Figure 4D summarize the expression levels of the markers for both WT iNK cells and the IL-15$^{+/+}$/TGFβR2$^{-/-}$ iNK cells. The differentiation process was highly efficient as evidenced by that both WT iNK cells and IL-15$^{+/+}$/TGFβR2$^{-/-}$ iNK cells have a population >95% CD45$^+$CD56$^+$, with a regular differentiation process used for our WT iNK cells. Additionally, both the WT iNK cells and the IL-15$^{+/+}$/TGFβR2$^{-/-}$ iNK cells expressed high levels of most of the NK activating receptors, such as Nkp30, NKp46, NKG2D, and DNAM1. Interestingly, the expression level of CD16 in IL-15$^{+/+}$/TGFβR2$^{-/-}$ iNK cells was higher than that of WT iNK cells.

Secretion of the soluble IL-15 was quantified by measuring IL-15 cytokine production at 24- and 72-h time points following medium change. As shown in Figure 4E, IL-15 was minimally expressed (0.2 pg/mL per 1E6 live cells at 24 h post-medium change; 2 pg/mL per 1E6 live cells at 72 h post-medium change) in supernatants collected from the WT of iNK cells at both time points. In contrast ($p < 0.001$), IL-15$^{+/+}$/TGFβR2$^{-/-}$ iNK cells produced soluble IL-15 at 9.68 pg/mL per 1E6 live cells (range 7.8–10.6) at 24 h post-medium change, and 80.16 pg/mL per 1E6 live cells (range 67.6–90.7) at 72 h post-medium change.

To further validate the KO of TGFβR2 protein in IL-15$^{+/+}$/TGFβR2$^{-/-}$ iNK cells, the cells were treated with bafilomycin A1, which prevents lysosome degradation and increases the expression level of TGFβR2.[21,22] Western blot assay results show that the cellular pools of TGFβR2 in the WT of iNK cells accumulated after 24 h treatment of bafilomycin A1 (Figure 4F, lanes 1 and 3). However, the expression of TGFβR2 could not be detected for IL-15$^{+/+}$/TGFβR2$^{-/-}$ iNK cells before or after the treatment of bafilomycin A1 (Figure 4F, lanes 2 and 4), confirming the true phenotypic KO of TGFβR2.

Functional characterization of the IL-15$^{+/+}$/TGFβR2$^{-/-}$ dual-edited iNK cells

To investigate whether the IL-15$^{+/+}$/TGFβR2$^{-/-}$ iNK cells could survive and proliferate in the NK expansion medium without

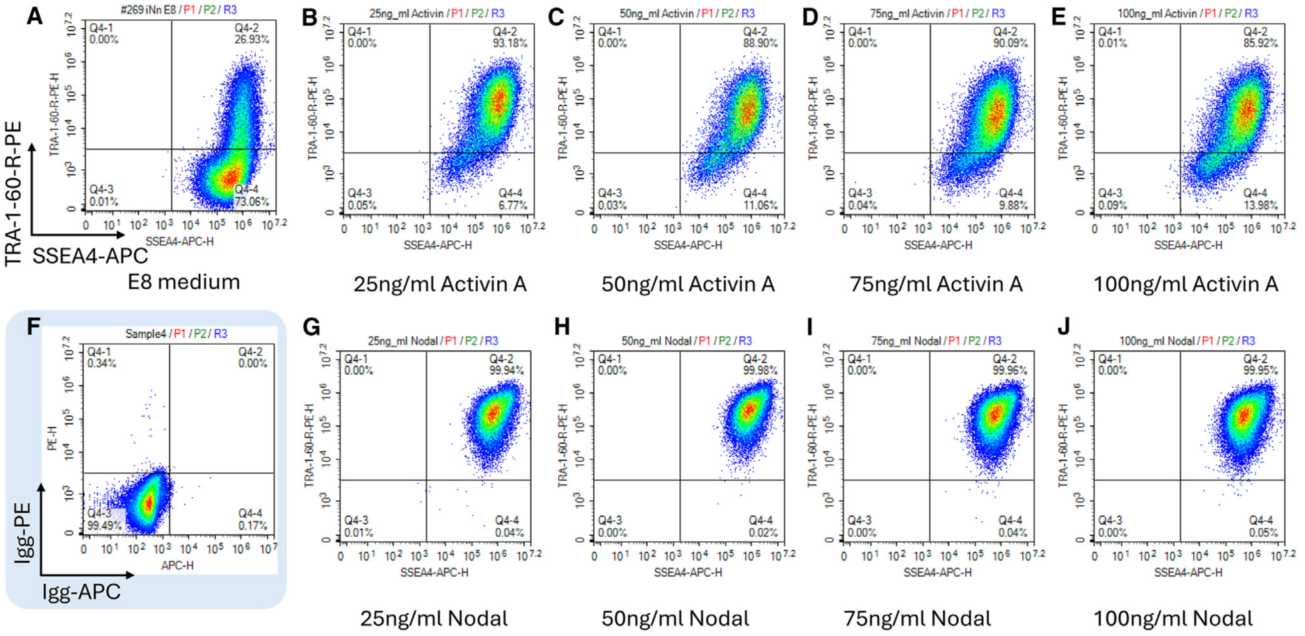

Figure 3. Pluripotency of iPSCs with TGFβR2 KO can be maintained in E8 medium supplement with Nodal

TGFβR2$^{-/-}$ iPSC cells were cultured in E8 medium (A) or in E8 medium supplemented with different concentrations of activin A (B–E), or Nodal (G–J) in a 37°C/5% CO_2 incubator. After 18 days of culture, TGFβR2$^{-/-}$ iPSC cells were collected and stained for pluripotency markers SSEA4 and TRA-1-60. The expression levels of these markers are shown by representative flow cytometry plots. The flow cytometry plots of Igg-PE and Igg-APC staining are also displayed (F).

IL-2 supplementation, we performed a cytokine-independent survival/growth assay (Figure 5A). Equal numbers of WT of iNK cells and IL-15$^{+/+}$/TGFβR2$^{-/-}$ iNK cells were plated in the NK expansion medium with or without IL-2. After 3 or 7 days of culture, only 1% of WT iNK cells remained viable. In contrast, the majority of IL-15$^{+/+}$/TGFβR2$^{-/-}$ iNK cells were able to survive without IL-2 supplementation, with their numbers increased by 50% from day 3 to day 7 in the NK expansion medium.

To investigate whether the TGFβR2 KO confers the cells' resistance to TGF-β suppressive signaling, we compared the phenotype and function of the WT of iNK cells and IL-15$^{+/+}$/TGFβR2$^{-/-}$ iNK cells with or without 5 days of TGF-β1 treatment. Flow cytometry results demonstrated that expression levels of the NK cell-activating receptors, including NKG2D, DNAM1, NKp30, and CD16, decreased in the WT iNK cells after TGF-β1 treatment (Figure 5B, left). In contrast, the expression levels of these markers were not affected in the IL-15$^{+/+}$/TGFβR2$^{-/-}$ iNK cells (Figure 5B, right) after exposure to TGF-β1. Interestingly, the surface level of NKp46 was not affected when the WT iNK cells were exposed to TGFβ1 (Figure 5B, left). Additionally, the expression of CD2, used as a control,[23] did not change in either the WT iNK cells or the edited iNK cells.

Finally, we examined the cytotoxicity activity of the iNK cells against Hep3B-GFP cells with or without the treatment of TGF-β1.[24] While the cytotoxicity of the WT of iNK cells at an effector-to-target (E:T) ratio of 5:1 was reduced by more than half (51.66% ± 6.10%) upon pre-culture with TGF-β1 (Figure 5C) in 4 h, IL-15$^{+/+}$/TGFβR2$^{-/-}$ iNK cells maintained their cytotoxicity activity. This suggests that these IL-15$^{+/+}$/TGFβR2$^{-/-}$ iNK cells could be functionally resistant to TGF-β-mediated suppression in hepatocellular carcinoma (HCC), where TGF-β plays a central suppressive role.[25] We further examined the cytotoxicity of the IL-15$^{+/+}$/TGFβR2$^{-/-}$ iNK cells against the K562-GFP target cells using a 48-h killing assay. This was compared with the WT iNK cells simultaneously. As shown in Figure 5D, the IL-15$^{+/+}$/TGFβR2$^{-/-}$ iNK cells show higher cytotoxic activity than that of WT iNK cells after 9 h. This superior cytotoxicity of IL-15$^{+/+}$/TGFβR2$^{-/-}$ iNK cells was maintained up to the end of the experiment, the 48-h mark.

DISCUSSION

Utilize cell lineage promoter to drive exogenous gene expression for iPSC-derived therapies

In this study, we present an improved approach for the generation of IL-15$^{+/+}$/TGFβR2$^{-/-}$ dual-edited iNK cells by TALEN. One of the significant modifications from traditional methods is the utilization of the endogenous B2M promoter to drive the expression of IL-15 in human iPSCs. Conventionally, exogenous genes were expressed under constitutive promoters, such as EF1α or CMV promoters, resulting in their constitutive expression. While no adverse effects of constitutively expressed IL-15 on the expansion and proliferation of human iPSCs have been reported and IL-15 is known to preferentially direct the differentiation of lymphoid-progenitor cells toward NK cells rather than T cells,[26] concerns remain that the constitutive expression of exogenous cytokines or other genes might override the endogenous regulatory mechanism, leading to unnatural expression patterns and potentially affecting the physiological behavior of human iPSCs. Therefore, cell lineage promoters with minimal or no expression in

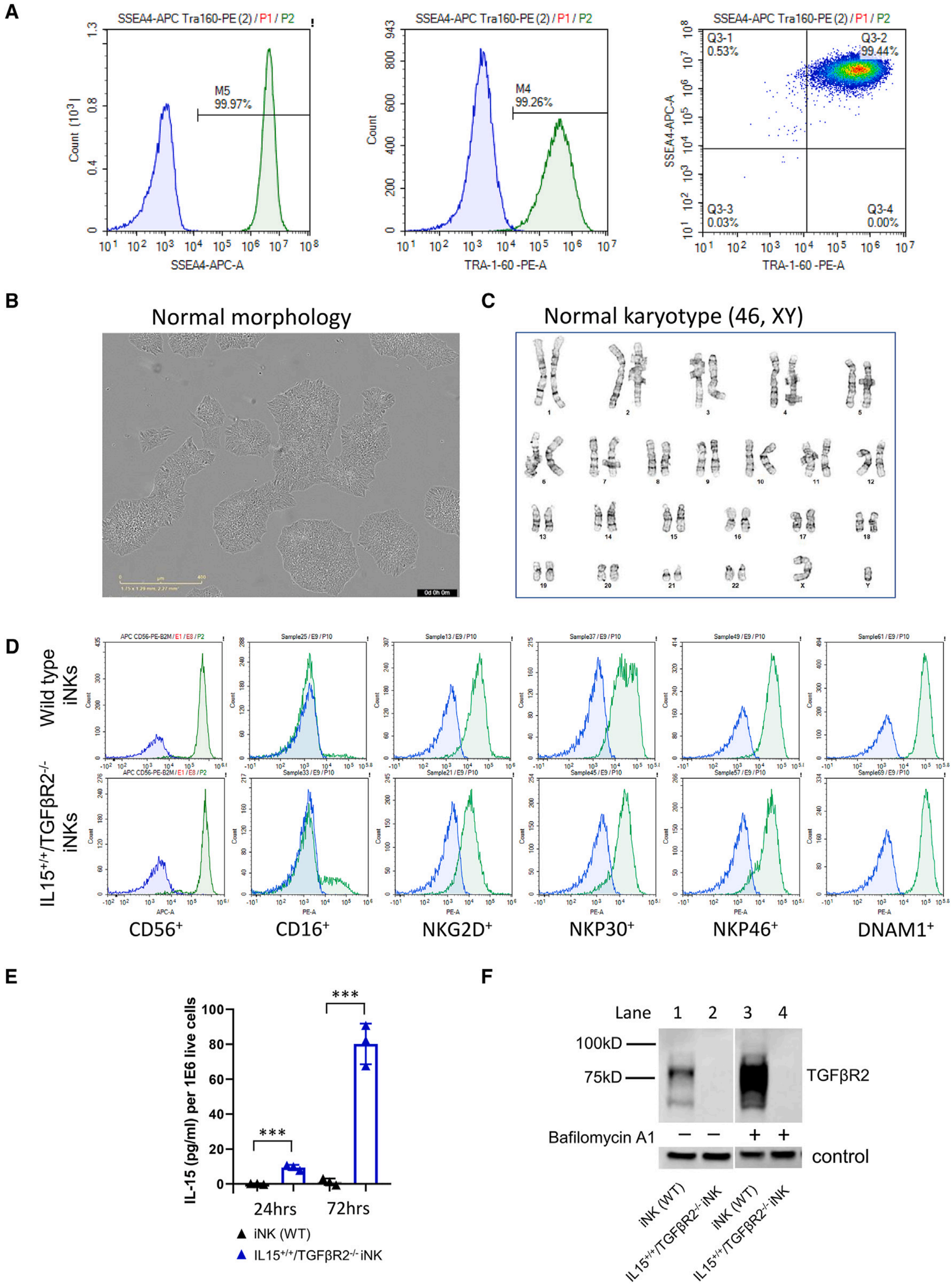

Figure 4. The IL-15$^{+/+}$/TGFβR2$^{-/-}$ iPSCs show normal morphology, normal karyotypes and pluripotency, and phenotypic characterization of the IL-15$^{+/+}$/TGFβR2$^{-/-}$ iNK cells

(A) Representative histogram figure and flow cytometry plots of pluripotency markers SSEA4 and TRA-1-60 for the IL-15$^{+/+}$/TGFβR2$^{-/-}$ iPSC single clone.

(B) A representative phase microscope image of the IL-15$^{+/+}$/TGFβR2$^{-/-}$ iPSC single clone. Scale bar: 400 μM.

(legend continued on next page)

iPSCs and robust expression in NK cells would be ideal. Our investigation into the expression patterns of three genes, GAPDH, B2M, and NKG2A, revealed that both B2M and NKG2A have minimal/no expression in iPSCs compared to GAPDH, while both exhibited significantly higher expression levels in iNKs, PBNKs, and CBNKs than in human iPSCs. However, B2M is expressed at much higher levels in NK cells than in NKG2A. Thus, we knocked in the soluble IL-15 under the control of the endogenous promoter of B2M. Given that B2M is also highly expressed in other immune cells, including $CD8^+$ T cells, regulatory T cells, $\gamma\delta$ T cells, monocytes, macrophages, and granulocytes,[27] its promoter can also be utilized to express exogenous genes for these various iPSC-derived immune cell therapies. However, other cell lineage promoters, such as the NKG2A promoter, NKp46 promoter, and NKG2D promoter for NK cells, can also be considered for expressing exogenous genes for iPSC-derived therapies. Depending on the desired expression level and expression pattern of the exogenous gene, one can carefully choose a most suitable promoter from these cell lineage promoters. It is worth noting that these cell lineage promoters may not have to be endogenous since many such promoters have been identified, such as promoters for NKp46 and B2M. Therefore, these promoters may be able to serve as exogenous promoters at a safe harbor site or at a target KO site to express the exogenous gene and achieve the same benefits.

Improved medium for TGFβR2 KO iPSC cells

The second optimization in our study is customizing the E8 medium for keeping the pluripotency of $TGF\beta R2^{-/-}$ iPSC clones. In the TME, TGF-β is abundantly present and is secreted by tumor cells, as well as several other cell types such as regulatory T cells, M2 macrophages, and myeloid-derived suppressor cells. This cytokine is well characterized as a potent suppressor of NK cell functions.[28] To make iNK cells more resistant to the suppressive TME, we decided to disrupt the normal TGF-β signaling pathway by knocking out the TGFβR2 gene.

The TGF-β signaling pathway plays a critical role in regulating pluripotency, self-renewal, and differentiation in human iPSCs. We observed that TGFβR2 KO iPSCs began to lose their pluripotency in E8 medium after 1 week. We also found that replacing the TGF-β in the E8 medium with recombinant human Nodal protein can maintain the pluripotency of TGFβR2 KO iPSCs for over 10 passages. Recently, some groups developed multiple gene-editing strategies such as $TGF\beta R2^{-/-}$/$CISH^{-/-}$ double KO[29,30] in NK cells to restore the antitumor immunity, or $TGF\beta R2^{-/-}$/$PD1^{-/-}$ double KO with the anti-mesothelin CAR in T cells for promoting long-term efficacy.[31] Our customized medium offers the possibility of applying these gene-editing strategies to iPSC-derived immune cell therapies and enables the production of MCB of such edited iPSC clones, paving the way for their clinical use.

TALEN-based iPSC-editing platform with high-QC analyses

In this study, besides the on-target gene-editing efficiency assay, morphology assay, karyotyping assay, and pluripotency assay, we identified candidate off-target sites for B2M_e1_1 TALEN and the TALEN TGFβR2_e3_2 TALEN in human iPSCs by GUIDE-seq. Importantly, the potential off-target editing was not observed by amplicon sequencing in those candidate off-target sites in the final products, the dual-edited $IL\text{-}15^{+/+}$/$TGF\beta R2^{-/-}$ iPSC clones. Thus, the B2M_e1_1 TALEN and the TALEN TGFβR2_e3_2 TALEN could be broadly used in other gene-editing studies related to B2M locus and/or TGFβR2 locus with less concern for any off-target editing.

In summary, we successfully developed an improved human iPSCs gene-editing platform using TALEN that can minimize any effect of the exogenous gene on iPSCs and allow the preservation of pluripotency of iPSC with TGFβR2 KO. The improvement can apply to other iPSC-derived cell therapies, such as iPSC-derived T cell therapies, and offers an opportunity to generate unlimited, homogeneous, and standardized cells for allogeneic off-the-shelf therapies.

Limitations of the study

There are limitations to our strategy and workflow. First, we displayed a non-destructive KI strategy on the B2M locus to preserve the endogenous B2M expression. While the expression of B2M in the edited iNK cells can reach $\sim$60%, it is not 100%. The decreased B2M expression can be explained by the general phenomenon that protein expression decreases at the second gene position when they are co-expressed in the bi-cistronic 2A construct.[32] This challenge might be overcome by designing a donor plasmid containing a full-length open reading frame of B2M in the first position in the bi-cistronic 2A construct while putting the soluble IL-15 or other exogenous gene(s) in the second position. Alternatively, one can target a site that is right after the B2M open reading frame and insert the sIL-15 there. In addition, B2M promoter can be used as an exogenous promoter to achieve the same benefits.

Second, how to distinguish a bona fide off-target was a common challenge. Although GUIDE-seq is considered the gold standard for investigating the candidate off-target sites, targeted amplicon sequencing can validate whether edits at these candidate off-target sites actually happen during gene editing. However, when the candidate off-target site is adjacent to any existing natural variant, it is challenging to validate/invalidate the true positive editing events in a noisy background. A co-analysis of edited and

(C) A representative karyotyping result of the $IL\text{-}15^{+/+}$/$TGF\beta R2^{-/-}$ iPSC single clone. High-resolution G-banding analysis showed that $IL\text{-}15^{+/+}$/$TGF\beta R2^{-/-}$ iPSC single clones have normal male karyotypes.

(D) WT iNK cells and $IL\text{-}15^{+/+}$/$TGF\beta R2^{-/-}$ iNK cells were stained for NK-specific marker and activating receptors. The expression of each marker is shown by representative flow cytometry plots.

(E) sIL-15 levels were measured by ELISA at 24 and 72 h for WT iNK cells and $IL\text{-}15^{+/+}$/$TGF\beta R2^{-/-}$ iNK cells. Error bars represent mean ± SD. ***$p < 0.001$ by Student's t test.

(F) WT iNK cells and $IL\text{-}15^{+/+}$/$TGF\beta R2^{-/-}$ iNK cells were treated with bafilomycin A1 for 24 h. Levels of TGFβR2 proteins pre-treatment and post-treatment were assayed by western blot with a loading control.

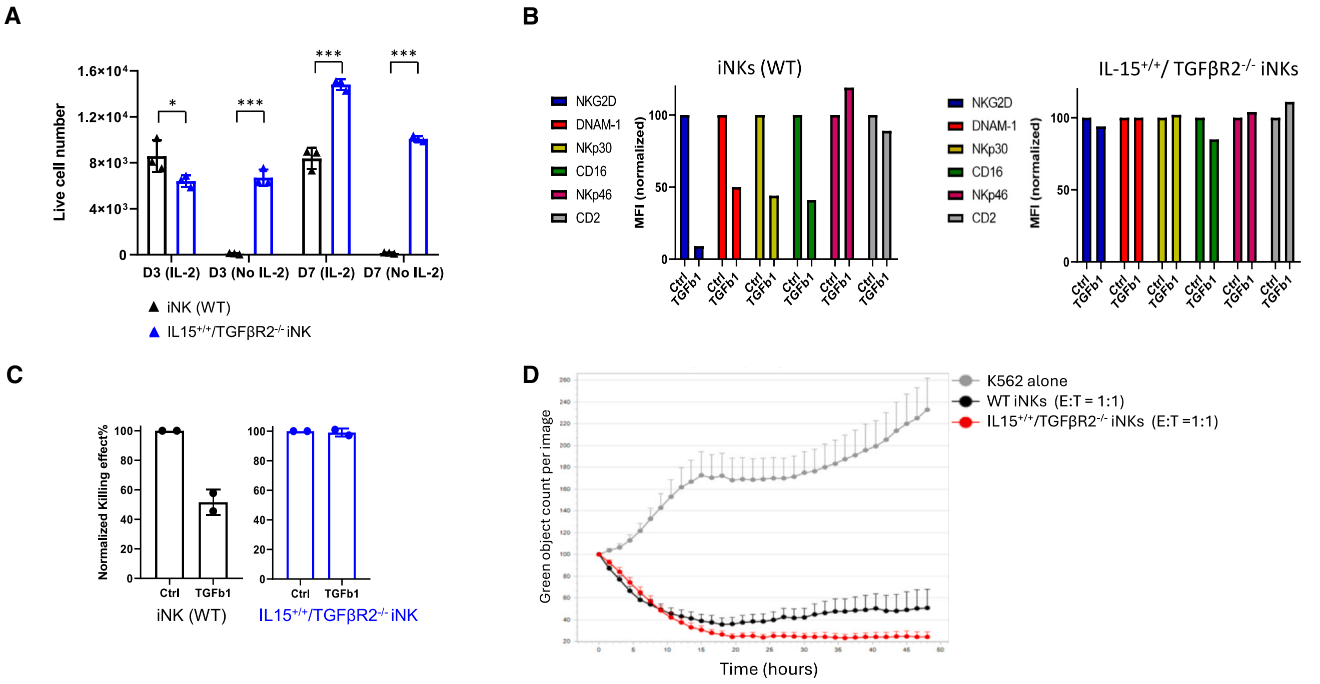

Figure 5. Functional characterization of IL-15$^{+/+}$/TGFβR2$^{-/-}$ iNK cells

(A) WT iNK cells and IL-15$^{+/+}$/TGFβR2$^{-/-}$ iNK cells were plated with the same number in NK expansion medium with or without IL-2. After 3 days (D3) or 7 days (D7), cells were collected and analyzed by flow cytometry with quantitative beads. Error bars represent mean ± SD. *$p < 0.05$ by t test; ***$p < 0.001$ by t test.

(B) WT (left) and IL-15$^{+/+}$/TGFβR2$^{-/-}$ iNK cells (right) were cultured under normal conditions (Ctrl) or in the presence of TGF-β1. The normalized MFIs of the staining of NK-activating receptors, including NKG2D, DNAM-1, NKp30, CD16, and NKp46, were measured by flow cytometry analysis. The expression level of CD2 was used as a control.

(C) The IL-15$^{+/+}$/TGFβR2$^{-/-}$ iNK cells are resistant to TGF-β-mediated suppression. The killing activity against Hep3B HCC tumor cells was compared between WT iNK cells and IL-15$^{+/+}$/TGFβR2$^{-/-}$ iNK cells under normal conditions (Ctrl) or in the presence of TGF-β1. Error bars represent mean ± SD.

(D) IL-15$^{+/+}$/TGFβR2$^{-/-}$ iNK cells were used as effectors at an E:T ratio of 1:1 with K562 myeloid leukemia cells. The non-edited iNK cells (WT iNKs) were used as control effectors, while K562 cells alone were used as reference. Data are representative of at least two independent experiments with each tumor cell line and are shown as means ± SDs (n = 6 technical replicates).

non-edited control samples might be helpful to remove existing background variants prior to genome editing.[33]

Lastly, while in this paper we demonstrated an improved approach to generate IL-15$^{+/+}$/TGFβR2$^{-/-}$ iNK cells and showed that the IL-15$^{+/+}$/TGFβR2$^{-/-}$ iNKs survive better in autonomous growth and are resistant to the suppressive TGF-β signaling, further characterization of these iNK cells is needed to better demonstrate the combined benefits of the two edits. For example, it will be important to explore the behavior of the dual-edited iNK cells in the presence of natural TGF-β suppression in more complex *in vitro* and *in vivo* models. Further *in vivo* efficacy and safety evaluations of these iNK cells are also warranted before moving into clinical development.

RESOURCE AVAILABILITY

Lead contact

Any additional information required to re-analyze the data reported in this paper is available from the lead contact upon request, Dr. Wei Li, wei.li@cytoviatx.com.

Materials availability

The donor plasmid used in this study can be shared by the lead contact with a completed materials transfer agreement.

Data and code availability

- Sequencing data and analysis in this paper will be shared by the lead contact upon request.
- This paper does not report original code.
- Any additional information required to reanalyze the data reported in this paper is available from the lead contact upon request.

ACKNOWLEDGMENTS

The authors thank D. Defranco, N. Zhang, and other research and development members at Cytovia Therapeutics for their help and support. This study was funded by Cytovia Therapeutics Inc.

AUTHOR CONTRIBUTIONS

Conceptualization, W.L., J.E., and D.T.; methodology, A.-P.C., P.G., L.L., P.A., H.H., H.-M.C., C.M., D.Z., A.A., J.E., and W.L.; validation, A.-P.C., P.G., L.L., P.A., and H.H.; investigation, A.-P.C., P.G., L.L., P.A., H.H., H.-M.C., D.Z., A.A., and W.L.; resources, A.-P.C., H.-M.C., D.Z., A.B., A.J., P.D., A.R., D.T., A.A., and W.L.; data curation, A.-P.C., P.G., L.L., P.A., H.-M.C., D.Z., A.A., and W.L.; writing – original draft, A.-P.C., D.Z., A.A., and W.L.; writing – review & editing, A.-P.C., P.G., L.L., P.A., H.-M.C., D.Z., V.A., A.B., A.J., P.D., A.R., D.T., A.A., J.E., and W.L.; supervision, W.L., A.-P.C., A.A., H.-M.C., and D.Z.; project administration, A.R., A.A., and W.L.; funding acquisition, D.T. and W.L. All authors have read and agreed to the published version of the manuscript.

DECLARATION OF INTERESTS

A.-P.C., P.G., L.L., P.A., H.H., H.-M.C., D.Z., A.R., D.T., A.A., and W.L. are employees of and hold stock or stock options in Cytovia Therapeutics. J.E. is a consultant to Cytovia Therapeutics and received funding support from Cytovia Therapeutics.

STAR★METHODS

Detailed methods are provided in the online version of this paper and include the following:

- KEY RESOURCES TABLE
- EXPERIMENTAL MODEL AND STUDY PARTICIPANT DETAILS
  - Culture of human induced pluripotent stem cells (iPSCs)
  - Culture of TGFβR2$^{-/-}$ iPSCs and IL15$^{+/+}$/TGFβR2$^{-/-}$ dual-edited iPSCs
  - Culture of human liver cancer cells (Hep3B) and K562 myeloid leukemia cells
- METHOD DETAILS
  - Quantification of B2M expression in human iPSCs and NK cells
  - Generation of TALEN mRNA and design of donor DNA
  - Nucleofections of human induced pluripotent stem cells
  - DNA extraction, PCR, surveyor nuclease assay and sequencing
  - Single cell cloning of electroporated human iPSCs
  - Karyotyping assay
  - GUIDE-seq experiments and bioinformatics analysis
  - Validation/invalidation of candidate off-target sites
  - Western blot analysis
  - Human IL-15 cytokine secretion analysis
  - Stepwise differentiation of iPSCs for NK development
  - Phenotyping of iPSC-derived NK cells
  - Cell number determination
  - Cytotoxicity assay of iNK cells
- QUANTIFICATION AND STATISTICAL ANALYSIS

SUPPLEMENTAL INFORMATION

Supplemental information can be found online at https://doi.org/10.1016/j.crmeth.2024.100857.

Received: November 9, 2023
Revised: May 7, 2024
Accepted: August 14, 2024
Published: September 10, 2024

REFERENCES

1. Truxova, I., Kasikova, L., Salek, C., Hensler, M., Lysak, D., Holicek, P., Bilkova, P., Holubova, M., Chen, X., Mikyskova, R., et al. (2020). Calreticulin exposure on malignant blasts correlates with improved natural killer cell-mediated cytotoxicity in acute myeloid leukemia patients. Haematologica *105*, 1868–1878. https://doi.org/10.3324/haematol.2019.223933.
2. Rakova, J., Truxova, I., Holicek, P., Salek, C., Hensler, M., Kasikova, L., Pasulka, J., Holubova, M., Kovar, M., Lysak, D., et al. (2021). TIM-3 levels correlate with enhanced NK cell cytotoxicity and improved clinical outcome in AML patients. OncoImmunology *10*, 1889822. https://doi.org/10.1080/2162402X.2021.1889822.
3. Menard, C., Blay, J.Y., Borg, C., Michiels, S., Ghiringhelli, F., Robert, C., Nonn, C., Chaput, N., Taieb, J., Delahaye, N.F., et al. (2009). Natural killer cell IFN-gamma levels predict long-term survival with imatinib mesylate therapy in gastrointestinal stromal tumor-bearing patients. Cancer Res. *69*, 3563–3569. https://doi.org/10.1158/0008-5472.CAN-08-3807.
4. Hoogstad-van Evert, J., Bekkers, R., Ottevanger, N., Schaap, N., Hobo, W., Jansen, J.H., Massuger, L., and Dolstra, H. (2019). Intraperitoneal infusion of ex vivo-cultured allogeneic NK cells in recurrent ovarian carcinoma patients (a phase I study). Medicine (Baltim.) *98*, e14290. https://doi.org/10.1097/MD.0000000000014290.
5. Geller, M.A., Cooley, S., Judson, P.L., Ghebre, R., Carson, L.F., Argenta, P.A., Jonson, A.L., Panoskaltsis-Mortari, A., Curtsinger, J., McKenna, D., et al. (2011). A phase II study of allogeneic natural killer cell therapy to treat patients with recurrent ovarian and breast cancer. Cytotherapy *13*, 98–107. https://doi.org/10.3109/14653249.2010.515582.
6. Handgretinger, R., Lang, P., and André, M.C. (2016). Exploitation of natural killer cells for the treatment of acute leukemia. Blood *127*, 3341–3349. https://doi.org/10.1182/blood-2015-12-629055.
7. Romee, R., Rosario, M., Berrien-Elliott, M.M., Wagner, J.A., Jewell, B.A., Schappe, T., Leong, J.W., Abdel-Latif, S., Schneider, S.E., Willey, S., et al. (2016). Cytokine-induced memory-like natural killer cells exhibit enhanced responses against myeloid leukemia. Sci. Transl. Med. *8*, 357ra123. https://doi.org/10.1126/scitranslmed.aaf2341.
8. Liu, E., Tong, Y., Dotti, G., Shaim, H., Savoldo, B., Mukherjee, M., Orange, J., Wan, X., Lu, X., Reynolds, A., et al. (2018). Cord blood NK cells engineered to express IL-15 and a CD19-targeted CAR show long-term persistence and potent antitumor activity. Leukemia *32*, 520–531. https://doi.org/10.1038/leu.2017.226.
9. Buren, L., Guo, C., Liu, D.F., Lazetic, S., and Trager, J. (2018). Co-expression of a chimeric NKG2D receptor with membrane bound IL-15 enhances natural killer cell function and long-term per-sistence in vitro and in vivo. Journal for ImmunoTherapy of Cancer *6*.
10. Goodridge, J.P., Mahmood, S., Zhu, H., Gaidarova, S., Blum, R., Bjordahl, R., Cichocki, F., Chu, H., Bonello, G., Lee, T., et al. (2019). FT596: Translation of First-Of-Kind Multi-Antigen Targeted Off-The-Shelf CAR-NK Cell with Engineered Persistence for the Treatment of B Cell Malignancies. Blood *134*, 301.
11. Woan, K.V., Kim, H., Bjordahl, R., Davis, Z.B., Gaidarova, S., Goulding, J., Hancock, B., Mahmood, S., Abujarour, R., Wang, H., et al. (2021). Harnessing features of adaptive NK cells to generate iPSC-derived NK cells for enhanced immunotherapy. Cell Stem Cell *28*, 2062–2075.e5. https://doi.org/10.1016/j.stem.2021.08.013.
12. Allen, A.G., Khan, S.Q., Margulies, C.M., Viswanathan, R., Lele, S., Blaha, L., Scott, S.N., Izzo, K.M., Gerew, A., Pattali, R., et al. (2024). A highly efficient transgene knock-in technology in clinically relevant cell types. Nat. Biotechnol. *42*, 458–469. https://doi.org/10.1038/s41587-023-01779-8.
13. Kao, C.Y., Mills, J.A., Burke, C.J., Morse, B., and Marques, B.F. (2023). Role of Cytokines and Growth Factors in the Manufacturing of iPSC-Derived Allogeneic Cell Therapy Products. Biology *12*, 677. https://doi.org/10.3390/biology12050677.
14. Ong, S.L.M., Baelde, H.J., van IJzendoorn, D.G.P., Bovée, J.V.M.G., and Szuhai, K. (2022). Identification of stable housekeeping genes for induced pluripotent stem cells and -derived endothelial cells for drug testing. Sci. Rep. *12*, 16160. https://doi.org/10.1038/s41598-022-20435-w.
15. Watabe, T., and Miyazono, K. (2009). Roles of TGF-beta family signaling in stem cell renewal and differentiation. Cell Res. *19*, 103–115. https://doi.org/10.1038/cr.2008.323.
16. Liao, N.S., Bix, M., Zijlstra, M., Jaenisch, R., and Raulet, D. (1991). MHC class I deficiency: susceptibility to natural killer (NK) cells and impaired NK activity. Science *253*, 199–202. https://doi.org/10.1126/science.1853205.
17. Tsai, S.Q., Zheng, Z., Nguyen, N.T., Liebers, M., Topkar, V.V., Thapar, V., Wyvekens, N., Khayter, C., Iafrate, A.J., Le, L.P., et al. (2015). GUIDE-seq enables genome-wide profiling of off-target cleavage by CRISPR-Cas nucleases. Nat. Biotechnol. *33*, 187–197. https://doi.org/10.1038/nbt.3117.
18. Shapiro, J., Iancu, O., Jacobi, A.M., McNeill, M.S., Turk, R., Rettig, G.R., Amit, I., Tovin-Recht, A., Yakhini, Z., Behlke, M.A., and Hendel, A. (2020). Increasing CRISPR Efficiency and Measuring Its Specificity in HSPCs Using a Clinically Relevant System. Mol. Ther. Methods Clin. Dev. *17*, 1097–1107. https://doi.org/10.1016/j.omtm.2020.04.027.

CellPress
OPEN ACCESS

19. Jo, S., Das, S., Williams, A., Chretien, A.S., Pagliardini, T., Le Roy, A., Fernandez, J.P., Le Clerre, D., Jahangiri, B., Chion-Sotinel, I., et al. (2022). Endowing universal CAR T-cell with immune-evasive properties using TALEN-gene editing. Nat. Commun. *13*, 3453. https://doi.org/10.1038/s41467-022-30896-2.

20. James, D., Levine, A.J., Besser, D., and Hemmati-Brivanlou, A. (2005). TGFbeta/activin/nodal signaling is necessary for the maintenance of pluripotency in human embryonic stem cells. Development *132*, 1273–1282. https://doi.org/10.1242/dev.01706.

21. Miller, D.S.J., Bloxham, R.D., Jiang, M., Gori, I., Saunders, R.E., Das, D., Chakravarty, P., Howell, M., and Hill, C.S. (2018). The Dynamics of TGF-beta Signaling Are Dictated by Receptor Trafficking via the ESCRT Machinery. Cell Rep. *25*, 1841–1855.e5. https://doi.org/10.1016/j.celrep.2018.10.056.

22. Bowman, E.J., Siebers, A., and Altendorf, K. (1988). Bafilomycins: a class of inhibitors of membrane ATPases from microorganisms, animal cells, and plant cells. Proc. Natl. Acad. Sci. USA *85*, 7972–7976. https://doi.org/10.1073/pnas.85.21.7972.

23. Ebert, E.C. (1999). Inhibitory effects of transforming growth factor-beta (TGF-beta) on certain functions of intraepithelial lymphocytes. Clin. Exp. Immunol. *115*, 415–420. https://doi.org/10.1046/j.1365-2249.1999.00824.x.

24. Dzieran, J., Fabian, J., Feng, T., Coulouarn, C., Ilkavets, I., Kyselova, A., Breuhahn, K., Dooley, S., and Meindl-Beinker, N.M. (2013). Comparative analysis of TGF-beta/Smad signaling dependent cytostasis in human hepatocellular carcinoma cell lines. PLoS One *8*, e72252. https://doi.org/10.1371/journal.pone.0072252.

25. Sung, P.S., and Jang, J.W. (2018). Natural Killer Cell Dysfunction in Hepatocellular Carcinoma: Pathogenesis and Clinical Implications. Int. J. Mol. Sci. *19*, 3648. https://doi.org/10.3390/ijms19113648.

26. Mrozek, E., Anderson, P., and Caligiuri, M.A. (1996). Role of interleukin-15 in the development of human CD56+ natural killer cells from CD34+ hematopoietic progenitor cells. Blood *87*, 2632–2640.

27. Uhlen, M., Fagerberg, L., Hallstrom, B.M., Lindskog, C., Oksvold, P., Mardinoglu, A., Sivertsson, A., Kampf, C., Sjostedt, E., Asplund, A., et al. (2015). Proteomics. Tissue-based map of the human proteome. Science *347*, 1260419. https://doi.org/10.1126/science.1260419.

28. Bellone, G., Aste-Amezaga, M., Trinchieri, G., and Rodeck, U. (1995). Regulation of NK cell functions by TGF-beta 1. J. Immunol. *155*, 1066–1073.

29. Moon, J.-I., Chin, M.S., Burden, A.T., Sexton, S., Wasko, K., Nasser, J.M., Antony, L.P., Wong, K.K., Borges, C.M., Morgan, R.A., and Welstead, G.G. (2020). Generation of Natural Killer Cells with Enhanced Function from a CRISPR/Cas12a-Edited Induced Pluripotent Stem Cell Line. Blood *136*, 8. https://doi.org/10.1182/blood-2020-139471.

30. Souza-Fonseca-Guimaraes, F., Rossi, G.R., Dagley, L.F., Foroutan, M., McCulloch, T.R., Yousef, J., Park, H.Y., Gunter, J.H., Beavis, P.A., Lin, C.Y., et al. (2022). TGFbeta and CIS Inhibition Overcomes NK-cell Suppression to Restore Antitumor Immunity. Cancer Immunol. Res. *10*, 1047–1054. https://doi.org/10.1158/2326-6066.CIR-21-1052.

31. Tang, N., Cheng, C., Zhang, X., Qiao, M., Li, N., Mu, W., Wei, X.F., Han, W., and Wang, H. (2020). TGF-beta inhibition via CRISPR promotes the long-term efficacy of CAR T cells against solid tumors. JCI Insight *5*, e133977. https://doi.org/10.1172/jci.insight.133977.

32. Liu, Z., Chen, O., Wall, J.B.J., Zheng, M., Zhou, Y., Wang, L., Vaseghi, H.R., Qian, L., and Liu, J. (2017). Systematic comparison of 2A peptides for cloning multi-genes in a polycistronic vector. Sci. Rep. *7*, 2193. https://doi.org/10.1038/s41598-017-02460-2.

33. Huang, L., Wang, D., Chen, H., Hu, J., Dai, X., Liu, C., Li, A., Shen, X., Qi, C., Sun, H., et al. (2023). CRISPR-detector: fast and accurate detection, visualization, and annotation of genome-wide mutations induced by genome editing events. J Genet Genomics *50*, 563–572. https://doi.org/10.1016/j.jgg.2023.03.010.

34. Flower, A., Chu, Y., Ayello, J., Kurtzberg, J., Lee, D., Cairo, M.S., and Cairo, M. (2016). Ex-Vivo Expansion of Previously Cryopreserved, Thawed and Re-Cryopreserved Cord Blood Mononuclear Cells (CBMC) Utilizing K562-mbIL21-41BBL; Potential for Adoptive Cellular Immunotherapy Post Umbilical Cord Blood Transplantation (UCBT). Immune Reconstitution *22*, S153. https://doi.org/10.1016/j.bbmt.2015.11.504.

STAR★METHODS

KEY RESOURCES TABLE

REAGENT or RESOURCE	SOURCE	IDENTIFIER
**Antibodies**		
APC anti-human SSEA-4	BioLegend	Cat# 330418; RRID:AB_2616819
PE anti-human CD3	BioLegend	Cat# 300441; RRID:AB_2562047
PE anti-human TRA-1-60-R	BioLegend	Cat# 330610; RRID:AB_2119065
PE anti-human β2-microglobulin	BioLegend	Cat# 395704; RRID:AB_2801053
PE anti-human CD226 (DNAM-1)	BioLegend	Cat# 338306; RRID:AB_2275498
PE anti-human CD337 (NKp30)	BioLegend	Cat# 325208; RRID:AB_756112
PE anti-human CD16	BioLegend	Cat# 302008; RRID:AB_314208
PE anti-human CD314 (NKG2D)	BioLegend	Cat# 320806; RRID:AB_492960
APC anti-human CD56 (NCAM)	BioLegend	Cat# 362504; RRID:AB_2563913
APC anti-human CD34	BioLegend	Cat# 343510; RRID:AB_1877153
PE anti-human CD45	BioLegend	Cat# 304008; RRID:AB_314396
PE Mouse IgG1, κ Isotype Ctrl	BioLegend	Cat# 400112; RRID:AB_2847829
PE Mouse IgG3, κ Isotype Ctrl	BioLegend	Cat# 401320; RRID:AB_10683168
PE Mouse IgG2b, κ Isotype Ctrl	BioLegend	Cat# 401208; RRID:AB_326637
Human beta 2-Microglobulin Antibody	R&D systems	Cat# MAB82481-100
Recombinant Anti-TGF beta Receptor II antibody	Abcam	Cat# ab184948; RRID:AB_2818975
**Chemicals, peptides, and recombinant proteins**		
Essential 8™ Medium	Gibco	A1517001
Essential 8™ Flex Medium Kit	Gibco	A2858501
TrypLE™ Select Enzyme	Gibco	12563011
APEL™2 Medium	STEMCELL Technologies	05270
Y-27632 ROCK inhibitor	STEMCELL Technologies	72302
Vitronectin	STEMCELL Technologies	07180
Ham's F-12 Nutrient Mix, GlutaMAX™ Supplement	Gibco	31765035
DPBS, no calcium, no magnesium	Gibco	14190144
Sodium selenate	MP Biomedicals	#194741
Ethanolamine	Sigma	#E0135-100mL
L-ascorbic acid	Sigma	#A4544-25G
2-mercaptoethanol	MP Biomedicals	#194705
BioLamina 521 CTG	BioLamina	CT521
DMEM, with GlutaMAX™ Supplement	Gibco	10569010
Ham's F-12 Nutrient Mix, GlutaMAX™ Supplement	Gibco	31765035
Fetal Bovine Serum	Gibco	10082147
GemCell™ U.S. Origin Human Serum AB, HI	GeminiBio	100–612
GlutaMAX 100X	Gibco	35050061
Gentamicin 10 mg/mL	R&D system	B20192
HEPES (1 M)	Gibco	15630080
MEM Non-Essential Amino Acids Solution (NEAA)	Gibco	11140050
IL-21	Peprotech	200–21
Flt3-L	Peprotech	300–19
IL-15	Peprotech	200–15
IL-3	Peprotech	200–03
IL-7	Peprotech	200–07
SCF	Peprotech	300–07

(*Continued on next page*)

Continued

REAGENT or RESOURCE	SOURCE	IDENTIFIER
BMP-4 Protein	PeproTech	AF12005ET
FGF basic	PeproTech	AF10018B
VEGF 165 Protein	PeproTech	10020
rHu IL-2, Liquid Syringe (1 mg)	Akron Biotechnology	AK9984-1000
CTS™ NK-Xpander™ Medium	ThermoFisher	A5019002
RIPA buffer	ThermoFisher	89900
LA Taq DNA polymerase	Takara	RR002M
Proteinase inhibitor	Sigma-Aldrich	P8340
TGFβ1	R&D Systems	7754-BH
Recombinant Human Nodal Protein	R&D Systems	3218-ND-025
Activin A	StemCell	78001
Critical commercial assays		
P4 Primary Cell 4D-Nucleofector X Kit L	LONZA	V4XP-4024
LentiSuite Deluxe Kit	System Biosciences	LV350A-1
Direct-zol™ RNA Miniprep kit	Zymo Research	R2050
Verso cDNA Synthesis Kit	ThermoFisher	AB1453A
TaqMan™ Fast Advanced Master Mix	ThermoFisher	4331182
Human IL-15 Quantikine ELISA kit	R&D systems	S1500
NEB HiScribe T7 ARCA kit	New England Biolabs	E2060S
NucleoSpin Tissue Genomic DNA Purification kit	Takara Bio	740952.50
Surveyor Mutation Detection Kit	IDTDNA	706020
Pierce Rapid Gold BCA Protein Assay Kit	ThermoFisher	A53225
SuperSignalTM West Pico	ThermoFisher	34580
Zombie NIR™ Fixable Viability Kit	BioLegend	423105
Invitrogen 123count eBeadsTM Counting Beads	ThermoFisher	01–1234
Roche Diagnostics KAPA HiFi PCR Kit	Roche	50-196-5207
Experimental models: Cell lines		
Human healthy donor iPSC line	New York Stem Cell Foundation	N/A
K562 cell line	ATCC	CCL-243
Hep3B cell line	ATCC	HB-8064
Oligonucleotides		
Primer B2M_F1: 5 gctgggcacgcgtttaatat 3′	Azenta/Genewiz	N/A
Primer B2M_R1: 5′ gccctaaactttgtcccgac 3′	Azenta/Genewiz	N/A
Primer TGFβR2_E1F1: 5′tcggtctatgacgagcagc3′	Azenta/Genewiz	N/A
Primer TGFβR2_E1R1: 5′gccccttgcaactgaacttt3′	Azenta/Genewiz	N/A
Primer TGFβR2_E3F1: 5′ tcaggaattcattggcaggct 3′	Azenta/Genewiz	N/A
Primer TGFβR2_E3R1: 5′ acatgcagagaacacccccta 3′	Azenta/Genewiz	N/A
Taqman primer for human B2M	ThermoFisher	Hs00187842_m1 (4331182)
Taqman primer for human GAPDH	ThermoFisher	Hs99999905_m1 (4331182)
Taqman primer for human NKG2A	ThermoFisher	Hs00970273_g1 (4331182)
Recombinant DNA		
Donor plasmid CYPL-001_pUC57	GenScript	U098EHC140-3/PD78723

(*Continued on next page*)

Continued		
REAGENT or RESOURCE	SOURCE	IDENTIFIER
Software and algorithms		
NovoExpress	Agilent	
GraphPad Prism 10 for Windows	GraphPad Prism	https://www.graphpad.com/updates
SnapGene 6.0	SnapGene	https://www.snapgene.com/updates/snapgene-6-0-0-release-notes
QuantStudio Data & Analysis v1.5.2	ThermoFisher	N/A
NovoExpress Flow Cytometry Software	Agilent	https://www.agilent.com/en/product/research-flow-cytometry/flow-cytometry-software/novocyte-novoexpress-software-1320805

EXPERIMENTAL MODEL AND STUDY PARTICIPANT DETAILS

Culture of human induced pluripotent stem cells (iPSCs)

The human induced pluripotent stem cells (iPSCs) were maintained in Essential 8 (E8) medium or Essential 8 Flex (E8-Flex) medium (Thermo Fisher Scientific, Waltham, MA, US) with its supplements in the vitronectin (StemCell Technologies, Vancouver, Canada) coated 6-well plate. The iPSCs were routinely passaged using TrypLE Select Enzyme at 37°C humidified incubator containing 5% CO_2 when reaching 60–80% confluent. The dissociated iPSCs were resuspended in three volumes of E8 medium with ROCK inhibitor Y27632 (StemCell Technologies, Vancouver, Canada). After 24 h, the medium was replaced by E8-Flex medium without the ROCK inhibitor, and subsequently, the medium was changed every other day.

Culture of TGFβR2$^{-/-}$ iPSCs and IL15$^{+/+}$/TGFβR2$^{-/-}$ dual-edited iPSCs

The TGFβR2$^{-/-}$ iPSCs and IL15$^{+/+}$/TGFβR2$^{-/-}$ dual-edited iPSCs were maintained in the Essential 8 Flex (E8-Flex) medium (Thermo Fisher Scientific, Waltham, MA, US) with its supplements and additional 100 ng/mL Nodal (R&D systems, Minneapolis, MN).

Culture of human liver cancer cells (Hep3B) and K562 myeloid leukemia cells

The human hepatoma cell line Hep3B and the human myeloid leukemia cell line K562 used in this study were purchased from ATCC (Manassas, VA, USA). Hep3B-GFP cells and K562-GFP cells were generated by stably transfecting GFP into Hep3B cells and K562 cells, respectively, using the LentiSuite Deluxe Kit (System Biosciences, Palo Alto, CA). The Hep3B and Hep3B-GFP cells were cultured in EMEM medium (Gibco, Life Technologies) supplemented with 10% FBS (Gibco, Life Technologies). The K562 and K562-GFP cells were cultured in RPMI medium (Gibco, Life Technologies) supplemented with 10% FBS (Gibco, Life Technologies). All the cells were maintained at 37°C in a humidified incubator with 5% CO2. The media was refreshed twice per week, and the cells were reseeded as necessary.

METHOD DETAILS

Quantification of B2M expression in human iPSCs and NK cells

To examine the mRNA expression level of B2M gene, total RNA was extracted from cultured cells using a Direct-zol RNA Miniprep kit from Zymo Research (Orange, CA) following the manufacturer's instructions. The quality of RNA samples was ascertained by measuring optical density (OD, 260/280) absorption. One microgram (1 μg) of purified total RNA was used to generate cDNA using Verso cDNA Synthesis Kit from ThermoFisher (Waltham, MA) in accordance with the manufacturer's manuals.

TaqMan real-time quantitative PCR amplification reactions were conducted using QuantStudio 5 Real-Time PCR Systems (Applied Biosystems). The real-time PCR was performed with the TaqMan Fast Advanced Master Mix for qPCR. Target gene expression assays (FAM-MGB) were ordered from ThermoFisher. Briefly, a 10-μL reaction mixture was prepared, consisting of 5-μL TaqMan Fast Advanced Master Mix, 1 μl TaqMan probe, and 1ul of cDNA template. The thermal cycle conditions were set as follows: 50°C for 2 min (UNG incubation), 95°C for 20 s (Taq activation), following 40 PCR cycles with 95°C for 1 s for denaturation, and 60°C for 20 s for annealing and extension. The relative quantification in gene expression was determined using the 2-ΔCt method. Using this method, we obtained the fold changes in gene expression normalized to an internal control gene -GAPDH.

Generation of TALEN mRNA and design of donor DNA

The TALEN plasmids encoding TALEN protein targeting the B2M exon1 (B2M_ e1_1), TGFbR2 exon1 (TGFβR2_e1_1) and exon3 locus (TGFβR2e3_1 and TGFβR2e3_2) were designed and produced using Cellectis' proprietary TALEN technology. The TALEN mRNAs were produced by *in vitro* transcription using the NEB HiScribe ARCA kit (New England Biolabs). The sequences targeted

by the four TALENs are the following (17 bp recognition sites, upper case letters, separated by a 13 or 14bp spacer with lower case letters):

B2M_e1_1: 5′ TTAGCTGTGCTCGCGCTactctctctttctGGCCTGGAGGCTATCCA 3′

TGFβR2_e1_1: 5′ TCGTCCTGTGGACGCGTatcgccagcacgatCCCACCGCACGTTCAGA 3′

TGFβR2_e3_1: 5′ TGTGTAAATTTTGTGATgtgagattttccaCCTGTGACAACCAGAAA 3′

TGFβR2_e3_2: 5′ TGATGTGAGATTTTCCAcctgtgacaaccagAAATCCTGCATGAGCAA 3′

To generate IL15$^{+/+}$/TGFβR2$^{-/-}$ iPSCs, an IL15-P2A expression cassette without stop codon was inserted at the B2M locus using the B2M_e1_1 TALEN and a linearized donor plasmid. To lead a high-level genome knock-in, the donor plasmid contains 800bp left and right homology arms which sequences match the upstream and downstream sequences of the start codon within the exon1 of B2M as shown in Figure 1. The coding sequence for IL-15 in donor plasmid was derived from the open reading frame of human IL15 (NM_000585). A self-cleaving P2A peptide in frame with the IL-15 and the endogenous B2M gene was also used. The IL15-P2A donor plasmid was synthesized by GenScript Biotech company (Piscataway, NJ, US).

Nucleofections of human induced pluripotent stem cells

The iPSC cells were co-transfected with TALEN mRNA and donor plasmid by 4D-Nucleofector X unit (Lonza, Basel, Switzerland) with CE-118 program using P4 Primary Cell 4D-Nucleofector X Kit. Electroporated iPSC cells were cultured in E8 medium with ROCK inhibitor Y27632 following by changing to E8-Flex medium without ROCK inhibitor 24 h later.

DNA extraction, PCR, surveyor nuclease assay and sequencing

Genomic DNA from iPSCs was isolated by using the NucleoSpin Tissue Genomic DNA Purification kit (Takara, San Jose, CA, USA). The PCR reactions were performed by LA Taq DNA polymerase (Takara, San Jose, CA, USA). To investigate the genome-editing efficiency for the TALEN, the targeted region of B2M exon1, TGFβR2 exon1 and exon3 were amplified, from the genomic DNA of the bulk electroporated iPSCs using the pair of primers B2M_F1: 5 gctgggcacgcgtttaatat 3′ and B2M_R1: 5′ gccctaaactttgtcccgac 3′, or the pair of primers TGFβR2_E1F1: 5′tcggtctatgacgagcagc3′ and TGFβR2_E1R1: 5′gccccttgcaactgaacttt3′, or the pair of primers TGFβR2_E3F1: 5′ tcaggaattcattggcaggct 3′ and TGFβR2_E3R1: 5′ acatgcagagaacacccta 3′, respectively. Then the amplified bands were purified and were analyzed by the Surveyor Mutation Detection Kit (IDT Biotechnology). The amplified ~400bp PCR bands were also sent for Amplicon EZ sequencing (Genewiz, South Plainfield, NJ, USA). To investigate the accuracy of knock-in cassette, the PCR amplified knock-in bands from genomic DNA of edited iPSC single colonies were purified, and were sent for Sanger sequencing (Genewiz, South Plainfield, NJ, USA) using the above primers B2M_F1 and B2M_R1. The alignments were performed by SnapGene software.

Single cell cloning of electroporated human iPSCs

The electroporated human iPSCs were cultured in the E8flex medium and dissociated with TrypLE for 7 min at 37°C once confluence reached 50%. The single-cell suspension was gently collected using two volumes of pre-warmed E8flex medium. The suspension was then centrifuged at 150rpm for 5 min. The supernatant was removed, and the pellet was resuspended by PBS and washed again. A 96-well flat-bottom plate was coated with 100μL/well Vitronectin (StemCell Technologies, Vancouver, Canada) for 1 h in a 37°C/5% CO_2 incubator. After aspirating the coating solution, 100μL/well of pre-warmed E8flex medium with Revitacell (ThermoFisher Scientific, Waltham, MA, US) was immediately added. The single-cell iPSC suspension was diluted in PBS to a concentration of 7,000 cells/ml. 700μL of the cell suspension was loaded into the sample chamber of a Hana (NamoCell, Mountain View, CA, USA) microfluidic chip. The prepared 96-well plate was loaded onto the plate stage of the Hana Single Cell Dispencer. In the Hana software, the trigger level was set as 100 in FSC, and the proper gate for FSC/SSC and FITC/PE was set. After removing the lid of plate, single cells were dispensed into the 96-well plate. After dispensing, the 96-well plate was covered with a clean lid and centrifuged at 100g for 1 min to accelerate the settlement of cells to bottom of plate. The plate was incubated in a cell culture incubator at 37°C/5% CO_2. The next day, 80μL E8flex medium with Revitacell was gently removed from each well, and 100μL fresh E8flex medium without Revitacell was added. The medium was changed every three days thereafter. The plate was observed under a microscope between days 7–14, and the wells with single clones were labeled. A 24-well plate was coated with Vitronectin for 1 h in a 37°C/5% CO_2 incubator, then the coating solution was replaced with 1mL/well E8flex medium with Revitacell. The single clone from the single well was dissociated with TrypLE for 7 min at 37°C. The cell suspension was then transferred into a new tube with two volumes of E8flex medium. After centrifugation, the pellet was resuspended in the E8flex medium with Revitacell. Half of the suspension cells were used for further genomic DNA extraction and characterization, and the other half were transferred into the prepared 24-well plate for further expansion. The medium was changed by E8flex without Revitacell every other day thereafter.

Karyotyping assay

The IL15$^{+/+}$/TGFβR2$^{-/-}$ iPSC cells from single colonies were cultured in the T-25 flask with our customized complete medium until at least 1 million cells in total. Then the T-25 flasks were wrapped and placed in separate proof zip-lock bags and were shipped in a small Styrofoam box at room temperature to Cell Line Genetics company for the G-banded karyotyping assay. Cytogenetic analysis was performed on twenty G-banded metaphase cells from the edited iPSC cell line.

GUIDE-seq experiments and bioinformatics analysis

Four aliquots of one million iPSC cells were electroporated with B2M_e1_1, TGFβR2_e1_1, TGFβR2_e3_1, or TGFβR2e3_2 TALEN, together with 100pmol double-stranded oligodeoxynucleotide (dsODN). The fifth aliquot of iPSC cells was electroporated with 100pmol dsODN only and was used as the negative control in the following GUIDE-seq analysis. Five electroporated iPSCs samples were cultured in the E8 medium for two days. The live iPSCs were harvested 48 h post-electroporation. After spinning down and removing the supernatant, the pellets of iPSCs were frozen at −80°C.

The GUIDE-seq experiments were performed by the GeneGoCell company (GeneGoCell, San Diego, CA, USA) with its standard process. Briefly, the genomic DNA was extracted from the iPSC pellet. By adding unique molecular identifier (UMI) tags during the PCR process, GUIDE-seq library construction could avoid the PCR bias and truly detect the on-target and off-targets quantitatively. The GUIDE-seq libraries were sequenced using Illumina NextSeq 2000 sequencer. Raw sequence reads were processed by the GeneGoCell NGS bioinformatics pipeline (v2.2.3). Low-quality reads were removed using quality score threshold 28 (Q28). The PCR duplicates were removed using UMI by GeneGoCell proprietary FASTQ file process tool. Reads were aligned to the human genome (hg38) using BWA v0.7.17-r1188. The integration junctions were identified for both the left and right ends of dsODN. The sequence surrounding the integration sites was extracted and analyzed for potential systemic noise, e.g., found in the negative control, low complexity region, and repetitive region. The alignment results were analyzed using G-GUIDE analysis program v3 to generate the genome-wide dsODN insertion sites.

Validation/invalidation of candidate off-target sites

The genomic DNA of two individual IL15$^{+/+}$/TGFβR2$^{-/-}$ dual-edited iPSCs single clones were extracted by NucleoSpin Tissue Genomic DNA Purification kit (Takara, San Jose, CA, USA). The frequency of insertion and deletion events (indels) generated at other candidate off-target sites were then quantitatively assessed using off-site-specific PCR and further Amplicon sequencing. The definition of background noise due to the non-negligible error rate of the Illumina sequencing process and the algorithm on calculation of the difference in indels frequency between TALEN-edited samples and the control samples were described before.[19] If the difference in indels frequency of the TALEN-edited iPSC and control parent non-edited iPSC samples was larger than the threshold (0.16%), the candidate off-target site was confirmed.

Western blot analysis

To investigate the TGFβR2 expression levels in the non-edited iNK cells and the IL15$^{+/+}$/TGFβR2$^{-/-}$ iNK cells, these cells were treated with or without 50nM Bafilomycin A1 (Merck Millipore, DMSO) for 24 h. Then the cells were collected, and the whole proteins were extracted by RIPA buffer plus proteinase inhibitor (ThermoFisher Scientific). The protein concentrations were measured by Pierce Rapid Gold BCA Protein Assay Kit. The iBlotTM2 Gel transfer system (ThermoFisher Scientific) was used to separate the proteins. The proteins were transferred to nitrocellulose membrane by using iBlotTM2 Dry Blotting System. The membrane was blocked by 5% non-fat milk in TBST overnight andwas incubated with 1st antibody – recombinant Anti-TGF beta Receptor II (Abcam, ab184948) for 2 h. The 2nd antibody was incubated for 1 h after 4 × 10min washed by 1xTBST. After another 4 × 10min washing with 1x TBST, the membrane was stained with SuperSignalTM West Pico Plus Chemiluminescent substrate.

Human IL-15 cytokine secretion analysis

The WT iNK cells and IL15$^{+/+}$/TGFβR2$^{-/-}$ iNK cells were cultured in the NK-Xpander medium in Grex-6M plate. The supernatant was collected either 24 h or 72 h after medium change for measuring of IL-15 secretion by the human IL-15 Quantikine ELISA kit (R&D systems, Minneapolis, MN) following the manufacturer's instructions.

Stepwise differentiation of iPSCs for NK development

The undifferentiated iPSCs were cultured in Essential 8 medium until the cells reached 50–70% confluent in the 6-well plate. The iPSC culture was washed twice with DPBS, and the iPSC clones were dissociated with TrypLE for 5–8 min at 37°C. Essential 8 medium was then added to the culture to dilute TrypLE. The dissociated iPSCs were harvested and transferred to 15mL tubes. After centrifugation, the cell pellet was resuspended in 3mL of APEL-2 medium to create a single cell suspension. An aliquot was taken for cell counting. Based on the cell count, 2.5E6 live cells were transferred to hematopoietic differentiation medium, consisting of APEL-2 medium supplemented with 50 ng/mL BMP4, 50 ng/mL VEGF, 50 ng/mL SCF, 20 ng/ml bFGF and 10uM Y27632. After thoroughly mixing the cells in the differentiation medium, 200uL of cell suspension was added to each well of a 96-well plate. The plate was centrifuged at 300g for 5 min before being transferred to 37°C/5% CO_2 incubator. After a 6-day incubation, the differentiated cells formed a spheroid in each well of the 96-well plate. The established spheroids were carefully transferred from seventeen wells of the well plate to each well of 6-well plate precoated with 10 ng/ml laminin 521 with the wide pore pipette tip. The spheroids were cultured in NK differentiation medium, consisting of a 2:1 mix of EMDM:F12 medium supplemented with 1x NEAA, 1x HEPES, 1x GlutaMax, 5 ng/mL sodium selenate, 50uM ethanolamine, 20ug/mL L-ascorbic acid, 1.1uM 2-mercaptoethanol, 0.1% gentamicin, 15% AB serum, 5 ng/mL IL3, 20 ng/ml IL7, 10 ng/mL IL15, 20 ng/mL SCF, and 10 ng/mL Flt3L. The culture was maintained in the 37°C/5% CO_2 incubator for one week before the medium was replaced with NK differentiation medium without IL-3. In the first two weeks, the differentiation medium was replaced once a week. In the third and fourth weeks, the medium was replaced twice a week. At the end of the third week, an additional supplement of 1x ITS solution was added to the culture medium. After four weeks of culture, the differentiated

CellPress
OPEN ACCESS

cells were harvested from the 6-well plate and transferred to a 50mL tube. After centrifugation at 300*g* for 5 min, the cell pellet was resuspended in the NK culture medium, consisting of complete NK-Xpander medium with 10% AB serum and 100IU/mL of IL-2. An aliquot of the well-mixed cell suspension was taken for cell counting. Based on the cell density, the live iNK cells were co-cultured with feeder cells, irradiated EK562 cells which were engineered to express IL21 and 41BBL,[34] at a 1:5 ratio in NK culture medium in G-Rex6M in the 37°C/5% CO_2 incubator for a week. The cell counting and the co-culture procedures were repeated on a weekly basis for an additional two weeks. After a total of three weeks of culturing, half of the NK culture medium was replaced by 50mL of fresh complete NK-Xpander medium supplemented with 10% AB serum, 500IU/mL IL2, 10 ng/mL IL15 and 10 ng/ml IL21. The cells were cultured for an additional three days before being harvested for phenotype and function analysis.

Phenotyping of iPSC-derived NK cells

The antibodies and related isotype controls used in this study are listed in the key resources table. The antibodies staining procedure was performed according to the manufacturer's protocols and the cells were resuspended in 200ul BD staining buffer in 96-well plate. Flow cytometry analyses were performed with NovoCyte Flow Cytometer (Agilent Technologies, Santa Clara, CA). Cells in the lymphocyte gate were used for analysis. A total of 50,000 events were collected for each sample and the generated data were analyzed by the NovoExpress software (Agilent Technologies).

Cell number determination

Invitrogen 123count eBeads Counting Beads (Thermo Fisher Scientific, Cat#01–1234) were added to cultures prior to flow cytometry analysis, along with Zombie NIR dye (BioLegend, Cat#423105) for dead cell exclusion. Total live cell counts were determined as a ratio of the counted live cells to beads.

Cytotoxicity assay of iNK cells

For 4-h cytotoxicity assay against Hep3B, the WT iNK cells or the edited iNK cells were co-cultured with Hep3B-GFP cells at an E/T ratio of 5:1 for 4 h. NK cell cytotoxicity activity was then measured by staining the cells with a Zombie NIR viability dye (BioLegend, San Diego, CA) followed by flow cytometry analysis. For the 48-h cytotoxicity assay, the wild type and edited iNK cells were co-cultured with K562-GFP cells at the E/T ratio of 1:1. The K562-GFP cells were counted with the IncuCyte S3 system. Images were captured by an ×10 objective lens at 1-h intervals. Tumor cell growth was quantified based on the green object count per image.

QUANTIFICATION AND STATISTICAL ANALYSIS

Data are presented as mean ± standard error of the mean (SEM). Statistical significance was determined by GraphPad Prism software (9) using Student's t test, one-way or two-way ANOVA as indicated and where appropriate adjusted for multiple comparison. p values <0.05 were considered statistically significant and indicated by * ($p < 0.05$), ** ($p < 0.01$), or *** ($p < 0.001$).

Cell Reports Methods, Volume *4*

Supplemental information

An improved approach to generate IL-15$^{+/+}$/TGFβR2$^{-/-}$ iPSC-derived natural killer cells using TALEN

An-Ping Chen, Peng Gao, Liang Lin, Preeti Ashok, Hongzhi He, Chao Ma, David Li Zou, Vincent Allain, Alex Boyne, Alexandre Juillerat, Philippe Duchateau, Armin Rath, Daniel Teper, Antonio Arulanandam, Hao-Ming Chang, Justin Eyquem, and Wei Li

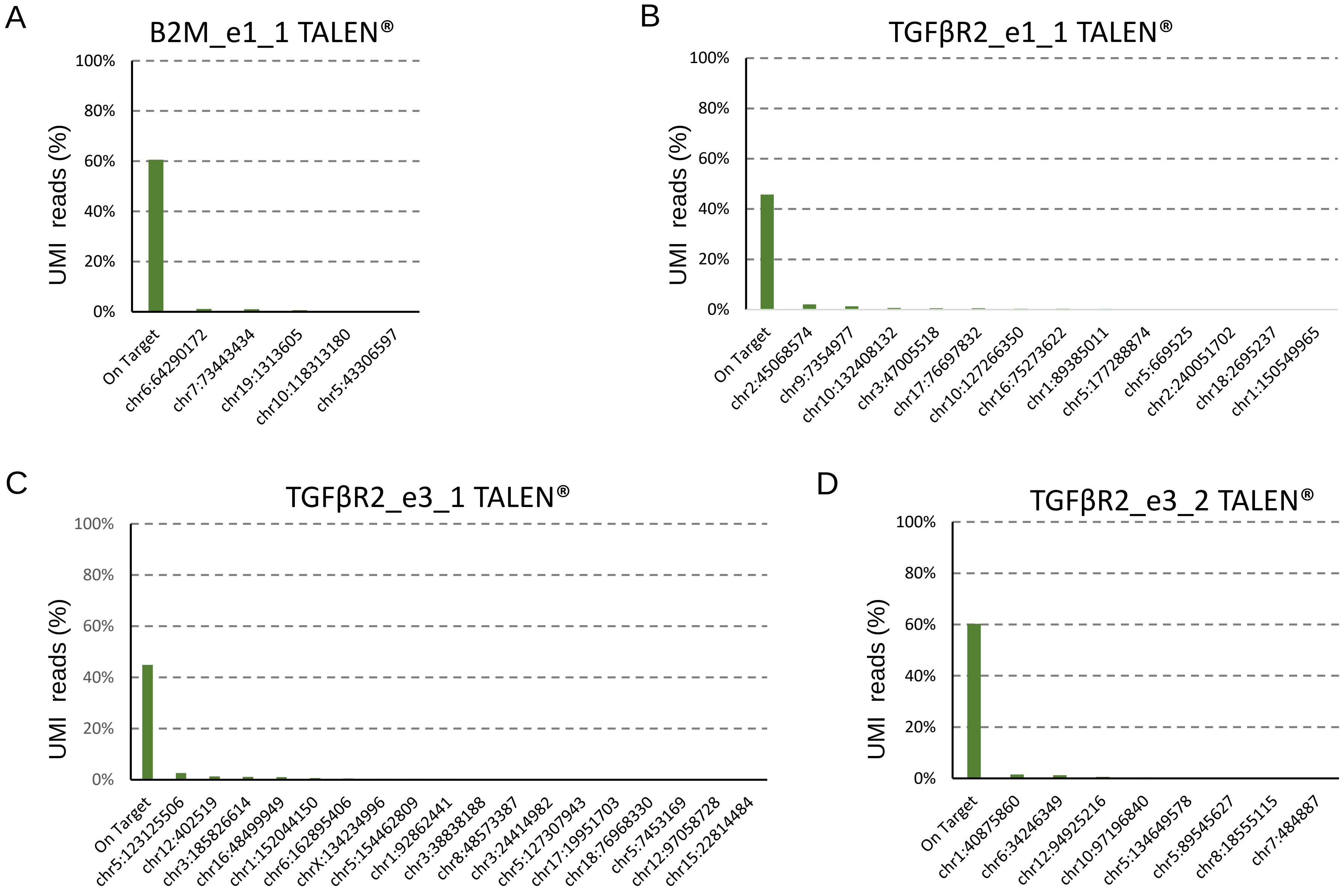

Figure S1: Potential candidate off-target site profiles of TALENs® targeting B2M and TGFbR2 loci in human iPSCs. Related to Table 1 and Table 2. Genome-wide potential candidate off-target sites for TALEN®s, B2M_e1_1, TGFβR2_e1_1, TGFβR2_e3_1 and TGFβR2_e3_2, in human iPSCs were analyzed by Guide-seq. The UMI (unique molecular identifier) reads percentage of the on-target site and potential candidate off-target sites for B2M_e1_1 TALEN® (A), TGFβR2_e1_1 TALEN® (B), TGFβR2_e3_1 TALEN® (C), and TGFβR2_e3_2 TALEN® (D) are displayed.

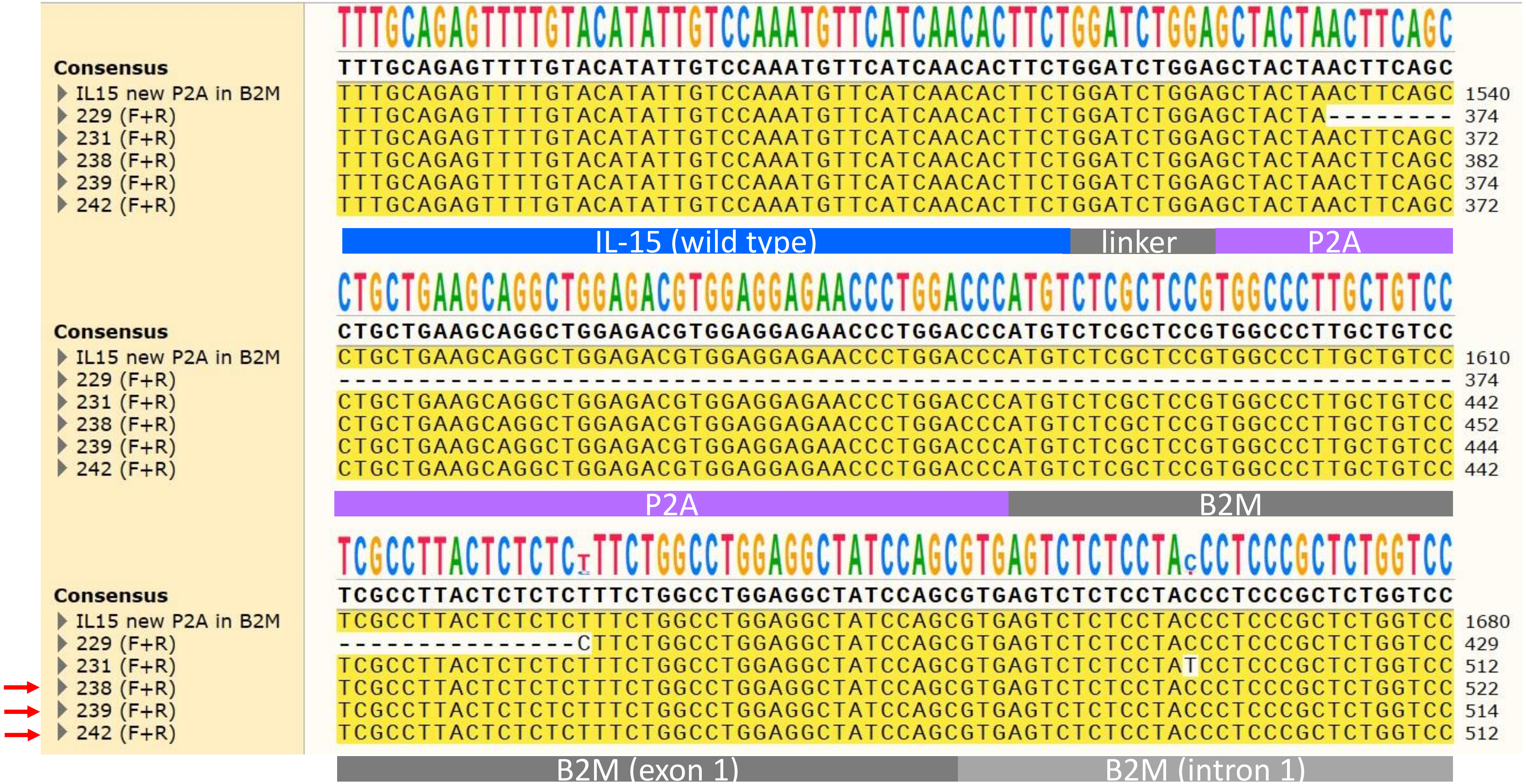

Figure S2: Precision of the insertion of IL15-P2A cassette at the *B2M* locus. Related to Figure 2. The PCR bands from five bi-allelic Knock-in iPSC clones (Figure 2F) were cut, purified, and sent for Sanger sequencing. Three of them (red arrow indicated) show desired knock-in sequence that encode the exogenous IL15 and the endogenous B2M exon1 in the same open reading frame (ORF).

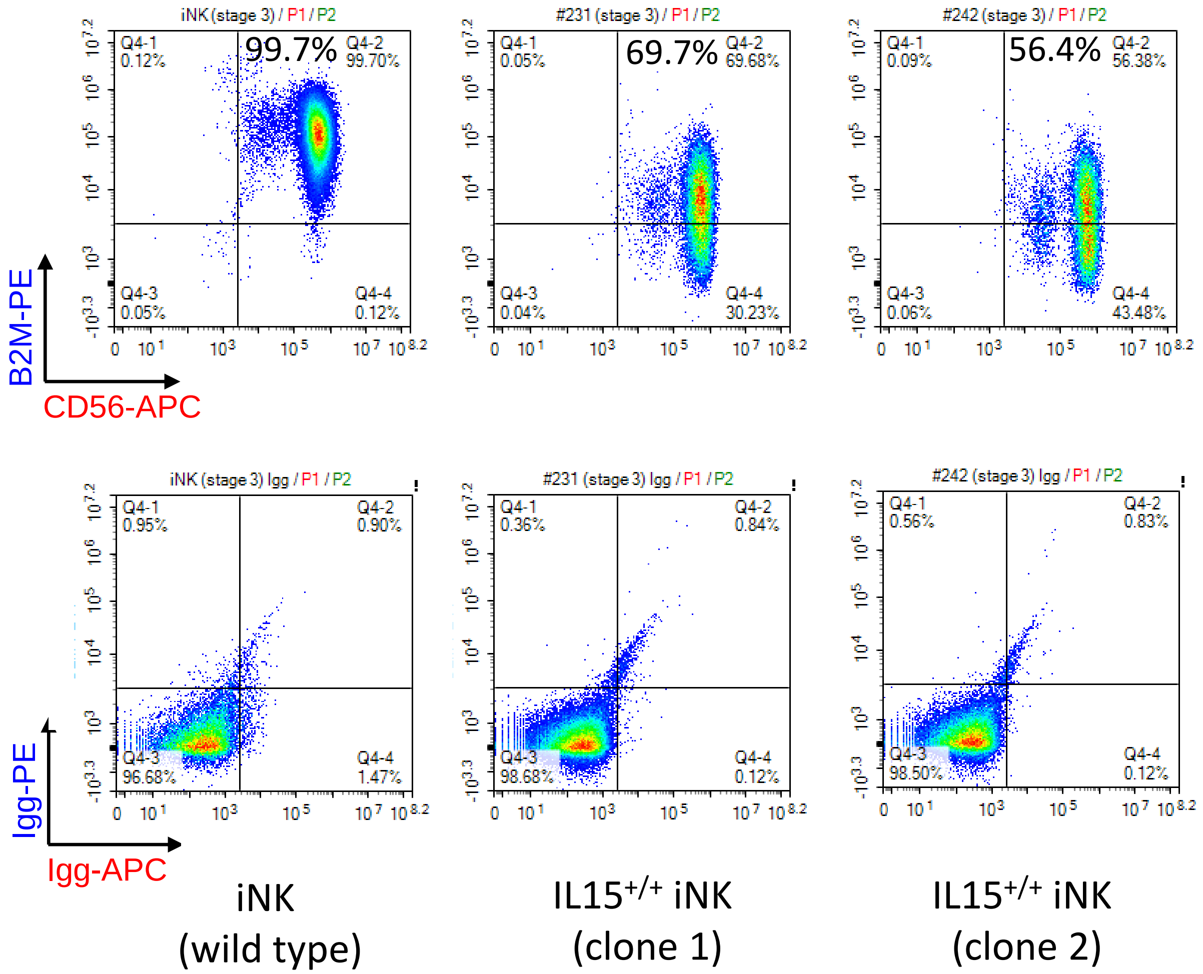

Figure S3: The expression of B2M were rescued in the IL15$^{+/+}$ iNK cells. Related to Figure 2. Flow-cytometry plots showing the surface expression level of B2M and CD56 in WT iNK cells and iNK cells from two IL15$^{+/+}$ iPSC clones (top panels). The flow-cytometry plots of control staining by Igg-PE and Igg-APC were also displayed (bottom panels).

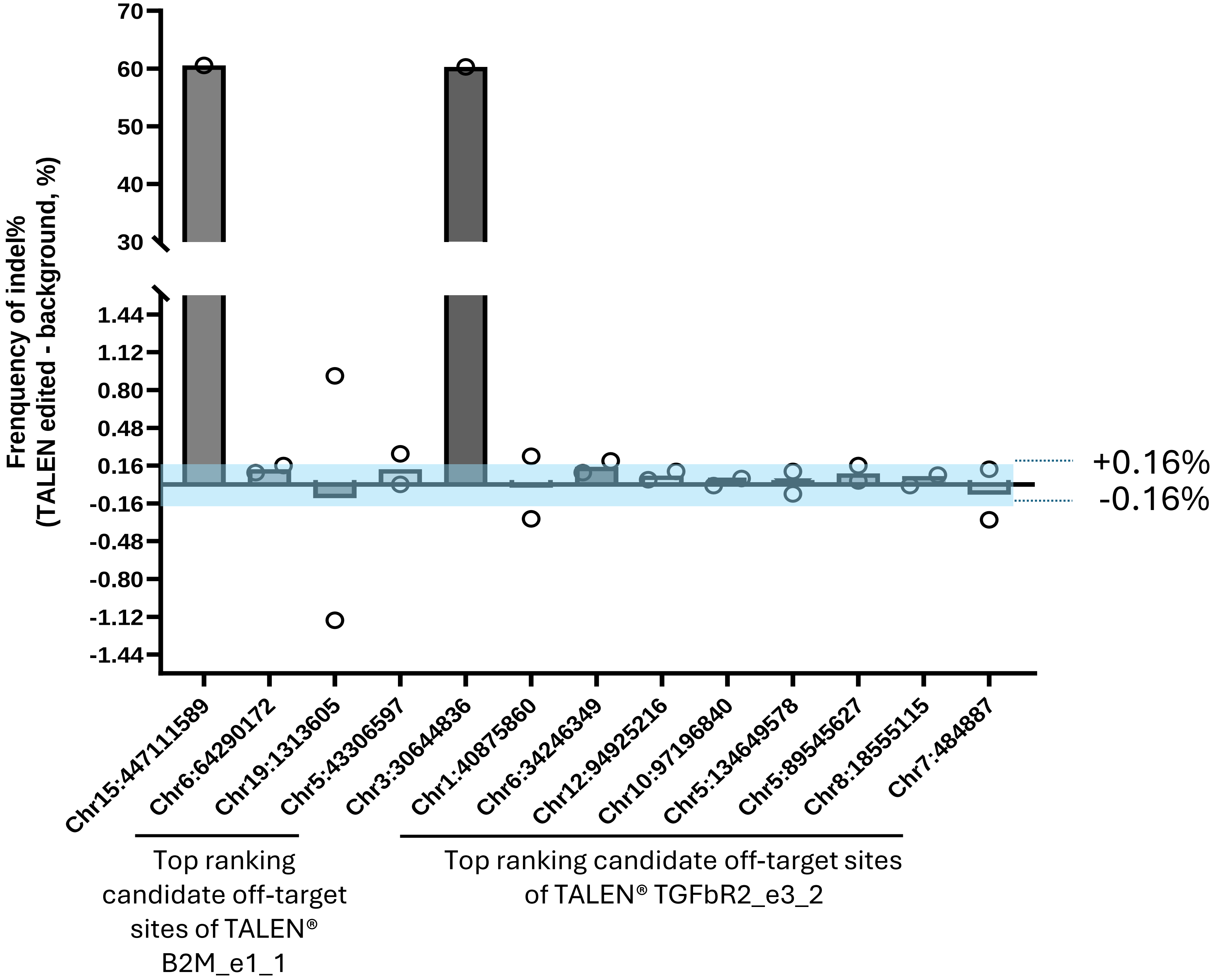

Figure S4: Validation/invalidation of potential candidate off-target sites in IL15$^{+/+}$/TGFβR2$^{-/-}$ iPSCs. Related to Table 1 and Table 2. Frequencies of insertion and deletions (indels) identified by Amplicon sequencing for the top candidate off-target sites of B2M_e1_1 and TGFβR2_e3_2 TALEN®s are displayed, except for two top-ranking candidate off-target sites of B2M_e1_1 TALEN® - chr7:73443435 and chr10:118313180 (see the explanation in the results). Frequencies of indels obtained in two individual IL15$^{+/+}$/TGFβR2$^{-/-}$ iPSC single clones subtracted from those obtained from the parent wild type iPSCs are indicated. The blue area indicates the data points falling within the threshold of significant indels detection (±0.16%). Frequencies of indels identified by GUIDE-seq for the on-sites of B2M_e1_1 and TGFβR2_e3_2 TALEN®s are also displayed in column 1 and 5.
